# Routinization, within-occupation task changes and long-run employment dynamics^☆^

**DOI:** 10.1016/j.respol.2022.104658

**Published:** 2023-01

**Authors:** Davide Consoli, Giovanni Marin, Francesco Rentocchini, Francesco Vona

**Affiliations:** a INGENIO (CSIC-Universitat Politecnica de Valencia), Spain; b Department of Economics, Society, Politics, University of Urbino 'Carlo Bo', Italy; SEEDS, Italy; FEEM, Italy; c European Commission, Joint Research Centre (JRC), Seville, Spain; d Department of Economics, Management and Quantitative Methods (DEMM), University of Milan, Italy; e University of Milan, Department of Environmental Science and Policy, Italy; f Fondazione Eni Enrico Mattei (FEEM), Italy; g OFCE Sciences-Po, France

**Keywords:** Tasks, Routinization, Technological change, Employment dynamics, Race between technology and education

## Abstract

The present study adds to the literature on routinization and employment by capturing within-occupation task changes over the period 1980–2010. The main contributions are the measurement of such changes and the combination of two data sources on occupational task content for the United States: the Dictionary of Occupational Titles (DOT) and the Occupational Information Network (O*NET). We show that within-occupation reorientation away from routine tasks: i) accounts for 1/3 of the decline in routine-task use; ii) accelerated in the 1990s, decelerated in the 2000s but with significant convergence across occupations; and iii) allowed workers to escape the employment and wage decline, conditional on the initial level of routine-task intensity. The latter finding suggests that task reorientation is a key channel through which labour markets adapt to various forms of labour-saving technological change.

## Highlights

• We capture within-occupation task changes over the period 1980–2010.
• We combine two data sources on occupational task content for the United States
• Within-occupation reorientation away from routine tasks accounts for 1/3 of the decline in routine-task use
• De-routinisation accelerates in the 1990s, decelerates in the 2000s with significant convergence across occupations;
• De-routinisation allows workers to escape employment and wage decline conditional on the initial level of routine-task intensity

## 1 Introduction

The task approach has become the main framework of reference to analyse both structural changes in labour markets (Autor et al., 2003; Acemoglu and Autor, 2011; Goos et al., 2014) and distributional implications (Autor et al., 2008; Lemieux, 2008). Classifying occupations by task content has proved particularly effective in explaining long-term labour market dynamics and in identifying the jobs that are more exposed to structural transformations such as technological change and globalization. However, the empirical literature that stems from this now prominent approach has so far focused primarily on changes at the ‘extensive’ margin, that is, reallocation of employment and wage differentials between occupations whose task content is held constant at some initial or average level. A framework in which job tasks are fixed is likely to lead to inaccuracies when it comes to capturing transformations of work activities and their implications for labour market outcomes, especially over extended periods. Conversely, a focus on within-occupation task changes has the potential to inform the design of training and education policies against the backdrop of the so-called race between technology and education (Goldin and Katz, 2010; Acemoglu and Autor, 2011; Vona and Consoli, 2015). Successful task reorientations are often identified as important mechanism to explain the resilience of aggregate employment in the face of rapid automation and other labour-saving structural transformations (Autor, 2015; Arntz et al., 2017; Dengler and Matthes, 2018).

Although the seminal study by Autor, Levy and Murnane (2003; henceforth ALM) calls attention to variations at the ‘intensive’ margin (i.e., changes in job tasks within an occupation), this dimension has remained relatively under-explored, also due to data limitations. The present study fills this gap by creating a time-varying measure of routine-task orientation for 322 occupations based on two main data sources for the United States (US), namely, the Dictionary of Occupational Titles (DOT) and its successor, the Occupational Information Network (O*NET). Such a measure allows us to build a consistent time series of within-occupation task changes over a thirty-year period, from 1980 to 2010, and to assess long-term structural changes in the US labour market over various phases of technological change.

Within-occupation task change is a long-term phenomenon that requires the mutual adaptation of demand and supply of skills. The paucity of suitable data sources offers a cue to the first and main contribution of the present paper. A thorough analysis of how job content evolves requires data that cover a long-time span. The most common resource to this end are the DOT, which was updated until 1991 upon the release of O*NET. Despite being designed for a similar purpose, however, matching these two data sources for extended time series analysis presents some challenges. Primarily, the complexity of the data has increased significantly with the inception of O*NET, so that using task items from these two sources, while maintaining consistency, requires a high degree of discretion on the part of researchers (Autor, 2013).

Section 2 puts forth a procedure to identify matching items in DOT and O*NET and construct a time-varying measure of job tasks for 322 occupations. The guiding criterion is the similarity between the task title and the task description. Based on this, we build an index of routine task intensity (RTI) that also accounts for within occupation task changes. We show that the proposed procedure reliably matches the moments of the distributions of the underlying task measures for each of the two data sources over time.

Section 3 presents descriptive evidence of changes in the task content of occupations based on the decennial Census and American Community Survey (ACS). Therein we show that within-occupation task changes account for 37 % of the overall decline in routine task use between 1980 and 2010. The within-occupation component is especially important in the 1990s (67 % of the decadal change), while it declines in the 2000s. Further, beneath the decrease of aggregate RTI we observe substantial change in the distribution of work tasks, in particular a catching-up of routine-task intensity during the last decade. Lastly, we find that changes in the task content of occupations are heterogeneous across sectors and broad occupational groups. The shift away from routine work of the 1990s was mostly in abstract occupations, and in non-manufacturing sectors, while the 2000s saw a reversal of this trend, with de-routinization being more prominent among blue collars and clerical occupations, and stronger in manufacturing.

Section 4 presents an exploratory analysis of the main correlates of within-occupation task changes. Therein, computer use at work in the 1990s exhibits a positive association with within-occupation changes in analytical and interactive tasks, and a negative association with changes in routine-task intensity. Further, within-occupation changes in RTI are associated with decennial employment growth over the entire timespan and especially in the 1990s even after controlling for, inter alia, initial routine task intensity. Finally, over the three decades under analysis, both employment and wages grow relatively faster in occupations that de-routine the most conditional on the initial level of routine task intensity. We interpret these regularities as empirical support to our proposed measure.

Our analysis contributes to various streams of research based on the task approach (ALM, 2003; Acemoglu and Autor, 2011). To begin with, the proposed approach affords the opportunity to analyse a time period that is both longer and more recent relative to prior studies on within-occupation task changes (Spitz-Oener, 2006; Ross, 2017, Ross, 2021). The most comprehensive study by Spitz-Oener (2006) focuses on Germany, but only until 1999 and not on long-term employment growth. We also add to prior work on new occupations and technology diffusion. While Lin (2011) tracks new job titles in US cities using the census classification based on the DOT over 1980–2000, we study the evolution of the task content of both existing and new occupations over 1980 and 2010 and find that task reorientation in existing occupations is more relevant in explaining labour market dynamics than that of new jobs.

Furthermore, the present study differs from closely related works which use job ad data from job vacancies (Atalay et al., 2020; Deming and Kahn, 2018). Although such an approach is promising in terms of accuracy in the construction of new task measures at both firm- and occupation-level, there are concerns regarding both the lack of adequate sample representativeness in building the task measures, especially for low-skilled workers, and the relatively short (Deming and Kahn, 2018) time spans available. The present study complements Atalay et al. (2020) in that, while their measure based on job vacancies better captures task change among abstract occupations, ours better captures task change among clerical and blue-collar jobs. In both Atalay et al. (2020) and our study, the main contribution is to propose new approaches to measure within-occupation task reorientation. Finally, our work adds to recent theoretical literature on task directed technical change. Acemoglu and Retrepo (2018) argue that the displacement effect of routine labour-replacing technology is counterbalanced by the emergence of new, more complex, high-skilled work activities. Our study offers a more nuanced view by showing that, while occupations that de-routinize the most exhibit positive employment dynamics, the bulk of within-occupation reorientation occurred among occupations with higher exposure to routine-replacing technological change, namely: middle-skill clerical and manual jobs. In other words, we show that adaptation through task reorientation occurs across the board. This is relevant for on-going debates on the aggregate effects of automation. To illustrate, Arntz et al. (2017) show that, even in a broad occupational category at high risk of automation a large fraction of jobs specializes in tasks that are complementary to the new capital equipment. Combined with our findings, this is again indicative that task reconfiguration is an important channel through which workers can cope with the labour market effects of technological change.

The remainder of the paper is organized as follows. Section 2 details the main sources and the procedures for the construction of our DOT-ONET database followed in Section 3 by descriptive evidence on the evolution of within-occupation task changes over the period 1980–2010. Section 4 presents exploratory analysis on the association between computer use at work and task configuration; on changes in education supply associated with shifts in occupational task content; and on employment outcomes by occupation and by macro-sectors. Section 5 summarizes and concludes.

## 2 Within-occupation task measures

### 2.1 Data sources

We combine information from different data sources to develop a consistent picture in the change of skill/task inputs over a thirty-year time period. In particular, we rely on the 1977 and 1991 editions (‘Fourth’ and ‘Revised Fourth’, respectively) of the DOT and the 2002 (version 4.0) and 2012 (version 18.0) editions of O*NET. Information on employment and educational attainment is retrieved from Census-based microdata, following recent literature (e.g., Autor and Dorn, 2013). We also use Integrated Public Use Micro Samples (IPUMS, Ruggles et al., 2018): for years 1980, 1990 and 2000 we use the 5 % sample of the decennial censuses, while for 2010 we combine three waves (2010, 2011, 2012) of the American Community Survey (ACS), which covers a representative sample of 1 % of the US population.

Combining these data sources, we build a balanced panel of 322 occupations based on the harmonized *OCC1990* occupational classification from IPUMS. This raises the issue of how to construct the task measures for the panel of 322 occupations aggregating information from DOT and O*NET, which are available at a much finer level of aggregation.^1^ ALM (2003) use weights of the April 1971 CPS Monthly File (National Academy of Sciences, 1981) and retrieve the employment shares of fine-grained job titles in the DOT for a single year (1971). This procedure, however, automatically eliminates the variation in within-task intensity associated with the emergence of new jobs with tasks that suit the demands of new technology (Lin, 2011). Since new jobs are important drivers of employment growth (Acemoglu and Restrepo, 2018), we follow Lin (2011) and use uniform weights to aggregate the task content of detailed occupational titles from DOT and O*NET to the level of the 322 occupations under analysis. In so doing, within-occupation task change also captures the emergence of new job titles and changes in the task content of the occupation.

### 2.2 Measure

The key variables for our analysis are measures of occupational skill requirements and task intensity. Previous studies have relied on one of the two sources available for the US, namely, the 1977 and 1991 editions of DOT (e.g., ALM, 2003) or O*NET (e.g., Acemoglu and Autor, 2011). One of the main contributions of the present paper is the elaboration of a novel matching procedure to merge DOT and O*NET for the purpose of extending the time span of the analysis.

The main critical issue is that O*NET has a comparatively higher number of task-related variables (approximately 400) compared to DOT (44). Moreover, O*NET measures have different scales: the ordinal ‘level’ scale (0–7) and the ordinal ‘importance’ scale (1–5).^2^ This is also recognised by Autor (2013, p. 192): “When the DOT was replaced by the O*NET in 1998, *the complexity of the database increased by an order of magnitude*. Version 14.0 of the O*NET database, released in June of 2009, contained 400 separate rating scales, which is almost half as many scales as the number of occupations coded by O*NET […] In practice, this means that *researchers who wish to use these databases as sources for task measures are essentially required to pick and choose among the plethora of scales available*, a problem that is much more severe for O*NET than for DOT.” [emphasis is our own]. Consequently, the task selection originally proposed by ALM (2003) is not suited to our purpose and, due to the constraints highlighted above (high dimensionality and plurality of scales in O*NET), researchers' discretion in the choice of task measures is critical.

As Ross, 2017, Ross, 2021 notes, although the rationale of O*NET was to generate a database with survey data collected solely from incumbent workers, the first release (version 4.0, June 2002) contained scores that were assigned by job analysts who used prior DOT versions as a reference. As a consequence, O*NET 4.0 blends the new rating system and the old methods. To check for measurement errors due to this that may affect the matching, we also tried O*NET 11.0 (December 2006) instead of O*NET 4.0. In O*NET 11.0 up to 647 occupations out of 798 (96.6 %) are assessed by means of survey data collected solely from incumbent workers.^3^ Since our main results are confirmed in the ancillary regressions based on O*NET 11.0, we maintain O*NET 4.0 to estimate decadal changes that are central to our analysis.^4^

Our proposed matching procedure follows three general rules. The first two concern the similarity in the *task title* and *task description*. Because O*NET was designed as the natural successor of DOT (Truthan and Karman, 2003), our main reference for the matching exercise is the summary of the DOT variables (occupations and work content) that have been converted to fit the relational model of O*NET as detailed in the first O*NET Data Dictionary (1998). Subsequent versions of the latter do not contain explicit references to DOT. Accordingly, we thoroughly examined variable descriptions in both sources to search for suitable matches. To illustrate, we consider that the DOT variable *Clerical Perception* (“the ability to perceive pertinent detail in verbal or tabular material. Ability to observe differences in copy, to proofread words and numbers, and to avoid perceptual errors in arithmetic computation”) bears a very similar title and description to the O*NET item *Clerical* (“knowledge of administrative and clerical procedures and systems such as word processing, managing files and records, stenography and transcription, designing forms, and other office procedures and terminology”).

The third general rule is maintaining *similar scales* for task scores in the two databases. Notably, we picked task measures with ordinal (Likert-type) scales. The problem here is that, while all O*NET task scores are defined on an ordinal scale, DOT assigns task scores using either an ordinal scale or dichotomous value. An example is “Direction Control and Planning” (DCP), which can either be present (equal to 1) or absent (equal to 0) in the DOT. Our choice to select items with similar scales avoids loss of information due to the transformation of ordinal variables into dichotomous ones and avoids manipulations that could alter the pattern of task changes through time.

Following these general rules, we identify suitable DOT items that correspond to the four dimensions of occupational task requirements identified by ALM (2003) as those that are particularly affected by automation and Information and Communication Technologies (ICTs): non-routine cognitive tasks (analytical and interactive), routine cognitive tasks and routine manual activity. Our first search yielded 16 different items, four for each of the dimensions of occupational task requirements that can be meaningfully associated between DOT and O*NET. In a second iteration we further reduced the selection to four items (one per dimension) following the aforementioned three criteria. These four items have been subsequently used to build an occupational task intensity measure.

Table 1 shows the DOT-O*NET matching items and reports the scale of each in the two data sources. When more than one candidate item was found in O*NET, we took the average value. To illustrate, for two measures the scale is similar (MANUAL and CLERIC, 1–5 level in DOT and 1–5 importance in O*NET) while it is different for MATH and LANGUAGE.^5^ The discrepancy is due to the different range between levels in DOT and levels in O*NET (DOT scale of 1–6 vs O*NET 0–7). Since the distribution of O*NET “level” is bounded, in most cases between 1 and 6, we truncate extreme values to 1 (bottom) and 6 (top).^6^ Consequently, we end up with 4 DOT variables linked to their corresponding O*NET match on similar scales.

**Table 1:** Match DOT-O*NET.

Task category (ALM, 2003)	DOT variable	DOT scale	O*NET variable	O*NET scale
Non-routine analytical	MATH: Mathematical development	1–6	Average of: - Mathematics (knowledge, 2.C.4.a, level) - Mathematics (skill, 2.A.1.e, level)	0–7 ↓ 1–6
Non-routine interactive	LANGUAGE: Language development	1–6	Speaking (skill, 2.A.1.d, level)	0–7 ↓ 1–6
Routine manual	MANUAL: Manual dexterity	1–5	Average of: - Manual dexterity (ability, 1.A.2.a.2, importance) - Arm-hand steadiness (ability, 1.A.2.a.1, importance)	1–5
Routine cognitive	CLERIC: Clerical perception	1–5	Clerical (knowledge, 2.C.1.b, importance)	1–5

Notes: Correspondence between the main DOT task categories used in ALM (2003) with O*NET task categories.

Subsequently, building on existing literature (Autor and Dorn, 2013; Goos et al., 2014) we combine matching DOT-ONET items into a normalised occupation-specific (*o*) and time-varying (*t*) index of routine intensity:

(1) $${\mathrm{RTI}}_{o,t}=\log\left(\frac{{\mathrm{CLERIC}}_{o,t}+{\mathrm{MANUAL}}_{o,t}}{{\mathrm{MATH}}_{o,t}+{\mathrm{LANG}}_{o,t}}\right)$$

The index captures the relative routine task requirements and, thus, the exposure to routine-replacing technical change of an occupation. Following the rationale of ALM (2003, p. 1287) we focus only on routine cognitive and routine manual tasks, and non-routine analytic and non-routine interactive tasks. In contrast to recent literature on the variation within the task content of occupations (Atalay et al., 2020; Ross, 2017, Ross, 2021), we employ an index of routine intensity. We prefer this to a single measure because the index can smoothen movements in task measures due to changes in scales and classification between DOT and O*NET and, thus, it is better suited to the analysis of long-term changes. Moreover, the index captures the relative importance of routine tasks relative to non-routine tasks, which is the key to assess the exposure of an occupation to routine-replacing technical change.

### 2.3 DOT-O*NET matching

With the aim of checking our matching choices from DOT to O*NET in the period 1990–2000 and the reliability of the proposed RTI index, we perform three exercises.

First, we search for marked differences between DOT and O*NET that may be attributable to our matching procedure. We did not find any systematic differences in average task scores between 1990 and 2000 (when O*NET was first introduced) compared to previous or subsequent periods (1980–1990 and 2000–2010). This is to say that, if systematic differences in the value of our task measures exist in blending DOT and O*NET, they are not necessarily due to our matching procedure. The quantile-to-quantile plots of Appendix A3 showing the distribution (by quantile) in the two selected years provide support to this.^7^ Even when some differences exist (e.g., Cleric and Manual in Figs. A3 and A4), they cancel each other out when we aggregate information for the four task measures into our routinisation index (Fig. A5).

Second, the result above is further corroborated by bootstrap-based tests on the first, second, third and fourth moments of DOT (1990) and O*NET (2000) distributions for our task measures (Table A3). Notably, we only find a significant difference between the averages for clerical between 1990 and 2000 and not for math, language and manual and the RTI index. A more variegated pattern emerges for other moments of the distributions (standard deviation, skewness and kurtosis). However, when statistically significant differences are detected between 1990 and 2000, the same is found for the following decade (2000−2010), meaning that changes in the distribution of task measures reflects a long-term pattern rather than a change that is artificially induced by our match.

Third, we compute the cross-sectional relationship between computer use at work and single items composing our task measures (Table A4).^8^ In line with expectations, computer use is significantly and positively correlated with abstract tasks (MATH and LANGUAGE) and routine cognitive tasks (CLERIC), while it is significantly and negatively correlated with (routine and non-routine) manual tasks (MANUAL and NRM) and with the RTI. Importantly, the magnitude of the estimated coefficients is similar across decades. Further analyses of the relationship between technology adoption and within-occupation task shifts in Section 4.1 reinforce this result.

Our matching procedure carries the major limitations due to differences between DOT and O*NET; the different versions within O*NET (particularly early job-analyst- vs survey-based versions); the choice of matched tasks between DOT and O*NET. So, results from our empirical exercise may well be biased due to the above, and should be taken with caution. Despite this, we believe that the three robustness exercises presented here are encouraging and mark a first step in an unexplored but arguably promising trajectory.

## 3 The evolution of within occupation task change over three decades

Before presenting some regularities based on our time-varying index of task change, Table 2 shows the trends in the use of human routine input in the US economy between 1980 and 2010. Therein, the evolution of routine task intensity captures both the within- and the between-component forces. In line with previous studies, the more general index of RTI used here shows that the overall level of routinization in 2010 is substantially smaller than that of 1980 (Column 1). The decline in RTI is very limited between 1980 and 1990 (only −2.2 %), accelerates remarkably in the 1990s (−10.7 %) and then, consistently with Beaudry et al. (2016), slows down again in the 2000s.

**Table 2:** Trends in RTI by macro-occupational group.

		All Occupations	Abstract	Clerical	Blue Collar	Services
1980	Mean	0.181	−0.322	0.193	0.438	0.353
	Std. dev.	(0.421)	(0.180)	(0.260)	(0.374)	(0.338)
	Emp. share	–	[0.234]	[0.277]	[0.341]	[0.234]
1990	Mean	0.158	−0.305	0.181	0.457	0.402
	Std. dev.	(0.438)	(0.188)	(0.276)	(0.425)	(0.320)
	Emp. share	–	[0.276]	[0.292]	[0.283]	[0.276]
2000	Mean	0.051	−0.482	0.117	0.449	0.285
	Std. dev.	(0.552)	(0.326)	(0.492)	(0.366)	(0.464)
	Emp. share	–	[0.302]	[0.279]	[0.259]	[0.302]
2010	Mean	0.010	−0.333	−0.001	0.277	0.292
	Std. dev.	(0.363)	(0.282)	(0.253)	(0.196)	(0.243)
	Emp. share	–	[0.327]	[0.259]	[0.219]	[0.327]
Average	Mean	0.091	−0.367	0.117	0.408	0.325
	Std. dev.	(0.456)	(0.273)	(0.351)	(0.361)	(0.349)
	Emp. share	–	[0.289]	[0.276]	[0.270]	[0.289]

Notes: Average RTI; . Weights used are the product of Census (1980; 1990; 2000) and ACS (2010) sampling weights and annual hours of labour supply.

The significant task change of the 1990s is consistent with the historical acceleration in the diffusion of ICTs (Autor et al., 1998). Looking at heterogeneous patterns across occupations, Abstract ones are the first to de-routinize in the first two decades, followed by Blue Collar and Clerical jobs in the last decade.^9^ This sequence of task reconfigurations is not only consistent with models of technological revolutions in which new technologies are adopted first by high-skilled workers and then by the least skilled ones (e.g., Zeira, 1998; Caselli, 1999; Beaudry and Green, 2005), but it also suggests that high-skilled workers have to learn new tasks that complement new technologies.

Changes to Abstract jobs in the third decade reveal the main limitation of our measure of routine task intensity compared to that used in related research by Atalay et al. (2020), namely that each component of the RTI index is bounded. Thereby, if an occupation had minimal level of routine intensity in 1980, a further decrease in the routine intensity cannot occur by construction. This is relevant for Abstract jobs that are near the minimum of routine task intensity.

### 3.1 Decomposing the long-term changes in routine task intensity

The trends shown in Table 2 pool together changes in the task content within each occupation as well as changes in the occupational composition. To gain a more precise understanding of the importance of within- vs between-occupation forces that have driven de-routinization, we decompose the overall change in RTI into three components:

(2) $$∆\mathrm{RT}I=\sum_{i,o}\left[\bar{\delta_{i}\phi_{i,o}}∆\mathrm{RT}I_{o}+\bar{\delta_{i}}∆\phi_{i,o}\bar{\mathrm{RT}I_{o}}+∆\delta_{i}\bar{\phi_{\mathrm{io}}\mathrm{RT}I_{o}}\right]$$

where *i* indexes industries and *o* occupations. $\bar{\delta_{i}\phi_{i,o}}\text{∆}\mathrm{RT}I_{o}$ represents the within-occupation component holding fixed both the within-industry *ϕ*_*i*, *o*_ and between-industry *δ*_*i*_ compositional changes.^10^ $\bar{\delta_{i}}\text{∆}\phi_{i,o}\bar{\mathrm{RT}I_{o}}$ is the between-occupation component and $\text{∆}\delta_{i}\bar{\phi_{\mathrm{io}}\mathrm{RT}I_{o}}$ is the between-industry component.^11^

Table 3 summarizes changes in job task input by intensive (within) and extensive (between) margins. The main takeaway is that the within-occupation component explains 37 % of overall decline in RTI over 1980–2010, while the between-occupation accounts for 40 % and the between-industry accounts for the remaining 23 %. Note that the within component closely tracks the overall evolution of the RTI index as its effect is concentrated in the 1990s, explaining 2/3 of the overall change in this decade. This contrasts with the weakening in the contributions to both the within-industry, between-occupation and the between-industry components in the 1990s.

**Table 3:** Decomposition of RTI.

	1980–1990	1990–2000	2000–2010	1980–2010
Within occupation	0.022	−0.072	−0.011	−0.062
Total between occupation	−0.032	−0.025	−0.011	−0.067
Total between industry	−0.018	−0.010	−0.009	−0.037
Total change	−0.028	−0.107	−0.031	−0.166

Notes: Decomposition of RTI based on Eq. (2). Weights used are the product of Census (1980; 1990; 2000) and ACS (2010) sampling weights and annual hours of labour supply.

Our finding on the prominence of the within-occupation component is consistent with the study by Spitz-Oener (2006) on the German labour market over the period 1979–1999. A recent paper by Atalay et al. (2020) also investigates changes in the task content of occupations in the US using textual data extracted from job ads published in major national newspapers. Remarkably, both studies find an acceleration in the within-occupation component in 1990s compared to the 1980s. Our analysis extends those studies by also including the 2000–2010 decade, where the significant deceleration of the within component closely matches that of de-routinization, which Beaudry et al. (2016) refer to as the ‘Great Reversal’.

### 3.2 Convergence and heterogeneity

**Fig. 1:**
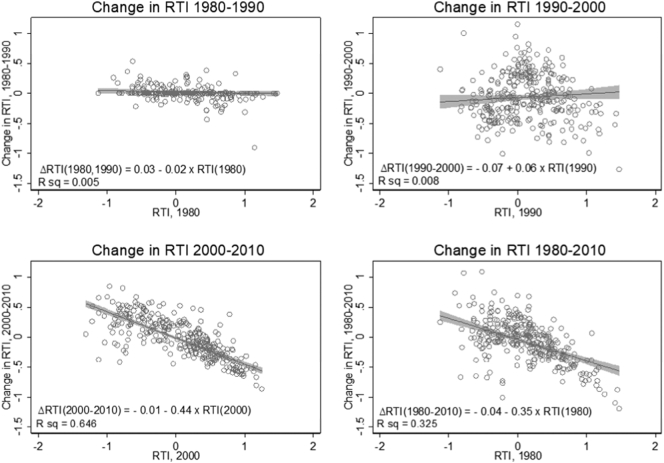
Convergence of RTI across occupations (*OCC1990*) Notes: Weights used in regressions are the product of Census (1980; 1990; 2000) and ACS (2010) sampling weights and annual hours of labour supply.

The scatter diagram in Fig. 1 illustrates the extent to which the routine task input of each occupation changes (vertical axis) relative to each occupation's initial RTI. Consistent with the above, we observe significant differences across decades. The flat, if slightly increasing, trends of the first two decades (top panels) contrast with the convergence signalled by pattern of the 2000s (bottom, left-hand panel). Therein, the decrease in routine task intensity is larger among jobs that had a higher RTI at the beginning of the period. On the whole, the pattern of the 2000s clearly dominates the overall change (bottom, right-hand side panel). Compared to the 1990s, where the distribution of routine task intensity to new technologies is slightly more dispersed, the 2000s are characterized by substantial redistribution of non-routine intensive tasks towards low- and medium-skilled occupations.

Table 4, Table 5 replicate the decomposition of routinization index by, respectively, macro occupational groups (Abstract, Clerical, Blue-collar and Service occupations) and the two macro-sectors (non-manufacturing and manufacturing). Consistent with this, the de-routinization observed in Table 2 is driven by a task reorientation primarily among Clerical and Blue-collar occupations. For Clerical occupations, the decline in the RTI is constant over time, while for Blue-collar occupations, it is more pronounced in the last decade.

**Table 4:** Decomposition of RTI by decade and occupational group.

	1980–1990	1990–2000	2000–2010	1980–2010
	Abstract			
Within occupation	0.026	−0.175	0.140	−0.009
Total between occupation	−0.002	−0.004	−0.004	−0.010
Total between industry	−0.002	0.003	0.009	0.010
Total change	0.022	−0.176	0.145	−0.009

	Clerical			
Within occupation	0.012	−0.050	−0.075	−0.113
Total between occupation	−0.031	−0.031	−0.006	−0.068
Total between industry	−0.007	−0.001	−0.001	−0.009
Total change	−0.026	−0.082	−0.082	−0.190

	Blue collar			
Within occupation	0.010	0.026	−0.170	−0.134
Total between occupation	0.005	−0.011	0.013	0.006
Total between industry	−0.002	−0.009	−0.001	−0.011
Total change	0.013	0.006	−0.158	−0.139

	Service			
Within occupation	0.068	−0.081	0.018	0.004
Total between occupation	−0.009	−0.023	0.009	−0.023
Total between industry	−0.005	−0.014	−0.001	−0.020
Total change	0.054	−0.118	0.026	−0.038

Notes: Decomposition of RTI based on Eq. (2). Macro-occupational groups defined in Table A2. Weights used are the product of Census (1980; 1990; 2000) and ACS (2010) sampling weights and annual hours of labour supply.

**Table 5:** Decomposition of RTI by decade and industry.

	1980–1990	1990–2000	2000–2010	1980–2010
	Manufacturing industries			
Within occupation	0.004	−0.054	−0.088	−0.138
Total between occupation	−0.048	−0.032	−0.031	−0.111
Total between industry	−0.012	0.003	−0.012	−0.021
Total change	−0.054	−0.083	−0.131	−0.270

	Non-manufacturing industries			
Within occupation	0.027	−0.076	0.003	−0.046
Total between occupation	−0.027	−0.023	−0.007	−0.057
Total between industry	−0.009	−0.008	−0.003	−0.012
Total change	−0.009	−0.107	−0.007	−0.123

Notes: Decomposition of RTI based on Eq. (2). Weights used are the product of Census (1980; 1990; 2000) and ACS (2010) sampling weights and annual hours of labour supply.

A recurrent pattern in our data is that during the first wave of ICTs in the 1990s the strongest change was the decline of RTI among Abstract occupations. Conversely, in the 2000s high-skill Abstract occupations became more routine intensive over time, again in line with the Great Reversal hypothesis (Beaudry et al., 2016). *Re*-routinization of Abstract occupations may reveal the greater capacity of machines in performing tasks such as translating complex documents, writing reports and legal briefs, as well as diagnosing diseases (Brynjolfsson and McAfee, 2014; Frey and Osborne, 2017), or simply a limitation of our measures for these occupations.^12^

When considering different industries (Table 5), the decline in the RTI is larger in manufacturing than in non-manufacturing ones. Moreover, within-occupation change contributes to more than half (51 %) of the total decline in RTI in manufacturing, while it only accounts for 38 % of the decline in RTI in non-manufacturing sectors. Importantly, the within-occupation component is relatively more important in non-manufacturing sectors (the 1990s) than in manufacturing sectors (the 2000s). This is consistent with the fact that the first wave of ICTs in the 1990s replaced clerical tasks in service sectors while the second wave in the 2000s affected the automation of manual tasks in industry (Brynjolfsson and McAfee, 2014). Together with the differential decadal patterns across occupations, we interpret this as a confirmation of the reliability of our proposed measure of within-task occupational changes to closely mimic well-established facts on labour market changes due to automation.^13^

### 3.3 A first glance at employment dynamics

**Fig. 2:**
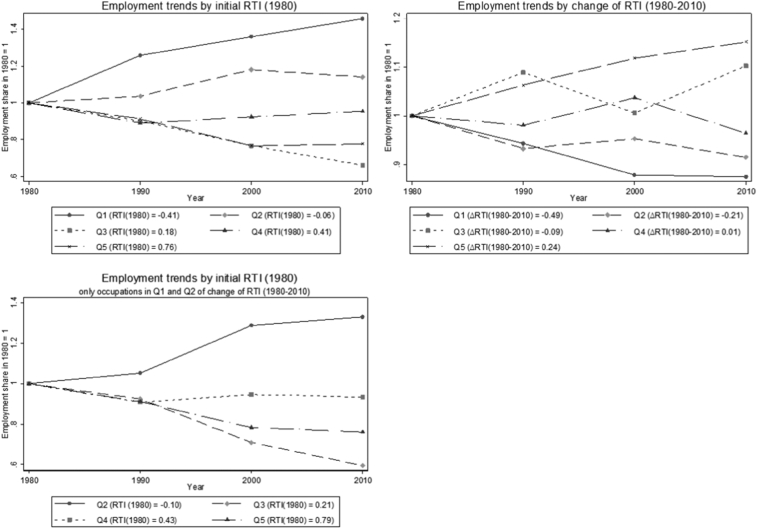
Trend in employment share by initial quintile of RTI or Δ RTI Notes: Trends in the annual hours of labour supply multiplied by sampling weights by groups of occupations defined as quintiles of the weighted distribution of RTI (1980, top-left and bottom-left panels) and of RTI change (1980–2010, top-right and bottom-left panels).

A key objective of the present study is to assess the relationship between qualitative change in the task content of occupations and changes in labour demand. To this end, we unpack aggregate trends of full-time US employees over the period 1980–2010 by partitioning the labour force into quintiles of initial RTI. In Fig. 2, the employment share of all groups is set to 1 in 1980 so that subsequent points in the diagram depict the mean employment of each group of occupations over time, net of overall employment growth. The first diagram of Fig. 2 (top, left-hand side) shows changes in employment by quintiles of initial values of RTI. Here, a divide emerges between occupations that were less intensive in routine tasks in the 1980 - which saw substantial increases in labour demand - and those with a stronger bias towards routine activities. This confirms a standard result of the existing literature: employment opportunities polarise depending on the initial level of exposure to routine-replacing technical change (ALM, 2003; Spitz-Oener, 2006; Deming and Kahn, 2018).

The second diagram of Fig. 2 (top, right-hand side) shows changes in employment by quintiles of within-occupational changes in RTI. Here we observe that occupations that de-routinize the most (Q1 and Q2) have worse employment performance throughout the period. This, however, does not account for the initial level of RTI. In the third diagram of Fig. 2 (bottom left-hand side), we unpack the trends of the sub-group of occupations that de-routinize the most – i.e., the first and second quantile in terms of change in routine task intensity – controlling for the initial level of RTI.^14^ We find that occupations that de-routinize the most are highly polarized in terms of long-term employment changes. Among those that are highly routine intensive (Q5 and Q4) we observe a large employment decline, while we observe large employment increases among those with low initial routine intensity (Q2^15^). That is, among the occupations that substantially de-routinize, only those that are initially less routine escape the employment decline.

Taken together, these stylized facts indicate that accounting for within-occupation changes in task content yields a complex picture of long-term structural changes in US labour markets. These issues will be explored more in detail in the next section with the aid of multivariate regressions to shed light on the conditional correlation between de-routinization and employment growth.

## 4 Drivers and implications of within-occupation task changes

Tasks are the key dimension of interest in the race between technology and education (Goldin and Katz, 2010; Acemoglu and Autor, 2011). On the one hand, new technologies have the potential to trigger radical reallocation in the share and type of tasks performed by humans, and this can occur along both the intensive (within-occupation) and the extensive (between-occupation) margin. On the other hand, where the intensive margin is relevant, educational programs are expected to keep pace with changes in the demand for specific tasks (Vona and Consoli, 2015). Since clerical jobs, such as clerks and assistants, have experienced substantial task reconfiguration towards organizational skills and non-routine tasks (∆*RTI* = −0.19 over the three decades), a concurrent change in training is necessary if workers are to be equipped with skills that match the incumbent technological paradigm. We expect that the diffusion of technology, such as ICTs, is the primary driver of within-occupation task shifts, and that these shifts are correlated with changes in the educational requirements. By testing these two predictions, the first two parts of this section closely follow related papers on changes in within-occupation task (ALM, 2003; Spitz-Oener, 2006).

The third and part of this section explores the correlation between within-occupation task shifts and labour market outcomes, namely employment and wages growth. Notice that task changes can be interpreted as a proxy of the degree of adaptation to structural transformations. Theory on routine-replacing technological change, such as the Ricardian model of Acemoglu and Autor (2011) and especially the recent extension with endogenous technical change of Acemoglu and Restrepo (2018), clearly advocates that successful adaptation should entail a reorientation away from routine tasks. Accordingly, occupations that de-routinize faster are expected to experience faster growth in wages and employment shares.

### 4.1 Technological change and within-occupation task changes

We examine the association between within-occupation task shifts and a proxy of technological change in the workplace: the change in the share of workers using computers. Although we are aware of the limitations of this, it is the only occupation-level measure for which data are available and that has been used in previous studies (e.g., Autor et al., 1998). As information on computer use at work by occupations from CPS (Current Population Survey) is only available for few selected samples, we focus our analysis on the 1990–2000 decade.^16^ This is critical for the present study given the data compatibility issues due to the matching of DOT and O*NET. Similarly, within-occupation task changes occurred mostly in this decade, which further reinforces our choice.^17^ We use a long-difference estimator to retrieve the associations between the change in the task content of occupation and the change in computer use, controlling for the initial levels of task input and computer use^18^:

(3) $$∆\mathrm{Tas}k_{o}^{1990-2000}=\alpha +\mathrm{\beta Tas}k_{o}^{1990}+\mathrm{\gamma Computer}\;\mathrm{us}e_{o}^{1989}+\delta ∆\mathrm{Computer}\;\mathrm{us}e_{o}^{1989-1997}+\varepsilon_{o}$$

Also, this analysis represents a further robustness check of choice of task items for the match between DOT and O*NET. For this reason, and in contrast to subsequent analyses where we focus on the aggregate routine-task intensity index, we present the correlations for the four component task items of the RTI index, the non-routine manual task measure and the RTI index itself.

Table 6 reports the results of this analysis. In line with the existing literature (ALM, 2003; Spitz-Oener, 2006), we find a positive contribution of the change in computer use to the within-occupation change in analytical (math) and interactive (language) tasks, a negative contribution to the change in routine (manual and clerical) and no clear effect on non-routine manual tasks (NRM). By combining these results, we find a negative association between the change in computer use and the change in routine-task intensity.

**Table 6:** Technological change and within-occupation task changes.

	(1)	(2)	(3)	(4)	(6)	(5)
Growth 1990–2000	Math	Language	Cleric	Manual	NRM	RTI
Task intensity in 1990	−0.436⁎⁎⁎	−0.389⁎⁎⁎	0.022	−0.238⁎⁎	−0.646⁎⁎⁎	−0.050
	(0.070)	(0.063)	(0.149)	(0.102)	(0.136)	(0.128)
Computer use in 1989	0.162⁎⁎	0.096	−0.013	−0.263⁎⁎⁎	−0.108	−0.068
	(0.081)	(0.086)	(0.122)	(0.061)	(0.068)	(0.255)
Growth in computer use (1989–1997)	0.146⁎	0.384⁎⁎⁎	−0.335	−0.429⁎⁎⁎	−0.101	−0.654⁎⁎
	(0.078)	(0.128)	(0.219)	(0.117)	(0.103)	(0.292)
R squared	0.327	0.209	0.0327	0.290	0.320	0.0392

Notes: *N* = 322 occupations. OLS regression. Weights used are the product of CPS Computer Use Supplement (October 1990) sampling weights and annual hours of labour supply. Robust standard errors in parenthesis. Computer use measured as the share of workers in the occupation that use computers on the job (source: CPS Computer Use Supplement October 1989, 1997). As a proxy for non-routine manual task intensity we use the DOT item EYEHAND (Eye-hand-foot coordination), that is measured over a range 1–5. This task is matched to the O*NET task ‘Response orientation’ (1.A.2.b.3, importance). See Table 1.

⁎ *p* < 0.1.

⁎⁎ *p* < 0.05.

⁎⁎⁎ *p* < 0.01.

Note that the increase in the share of workers using computers at work was 14.3 % during this decade. This implies an average change in RTI that is 1.6 times the actual change (−0.094 vs. −0.058). Although this may appear surprisingly large, it is in line with the estimates by ALM (2003; for the period 1984–1997 computerization more than fully accounts for the observed changes in single task measure) and Spitz-Oener (2006); effects ranging between 47 % (non-routine interactive) and 90 % (routine cognitive) that combined together in a RTI index will deliver an association of a similar size).

Overall, our data-set built on the match of DOT and O*NET confirms the strong association between computer use and task reconfiguration within an occupation. In light of this, we can safely attribute the bulk of this reconfiguration to routine-replacing technological change.

### 4.2 Implications of within-occupation task changes for long-term employment growth

We retrieve the conditional association between decennial employment growth and task reconfiguration by estimating the following equation^19^:

(4) $$∆\ln\left(L_{\mathrm{oi},t}\right)=\alpha +\beta_{1}\mathrm{RT}I_{o,t-1}+\beta_{2}∆\mathrm{RT}I_{o,t}+\gamma {\mathrm{NRM}}_{o,t-1}+\varphi {\mathrm{OFF}}_{o}+\delta_{t}+\delta_{i}+\varepsilon_{o,t}$$

where the decennial (or the thirty year) change in the log of employment of occupation *o* in industry *i* (*L*_*oi*, *t*_ ) is regressed on three lagged measures of occupational task orientation at the beginning of the period: i) *RTI*_*o*, *t*−1_, the initial value of routine intensity; ii) *OFF*_*o*_, a time-invariant index of offshorability as defined in Acemoglu and Autor (2011), iii) *NRM*_*o*, *t*−1_, non-routine manual intensity, as well as industry and year dummies. Controlling for offshorability and *NRM* is the most obvious and direct way to isolate the incidence of routinization from that of other intervening factors at the occupation-level.^20^ Our variable of interest is the long-term change in routine task intensity within an occupation, ∆*RTI*_*o*, *t*_.

Similar to Goos et al. (2014), estimates are performed at the occupation-by-industry level to control in a flexible way for industry-level drivers such as globalization that may influence employment change. As a variant of the long-term 30-year model, we also estimate Eq. (5) where all decades are stacked together.^21^ Finally, all estimates are weighted for the hours worked at the beginning of the period and the standard errors are clustered at the occupation-level.

An obvious caveat is that the coefficients of our main variable of interest, ∆*RTI*, cannot be interpreted as a causal effect, although the sources of estimation bias are likely to offset each other. On the one hand, self-selection of more skilled workers into occupations that de-routinize faster makes these occupations more productive and less likely to experience a decline, thus bringing an upward bias in the estimated coefficient of ∆*RTI*. On the other hand, the fact that the changes in *RTI* are bounded from above may lead to underestimating task changes in abstract occupations. Since the latter are becoming more complex - and are branded as ‘winners’ in terms of earnings and employment growth (Deming, 2017) - this would yield an underestimation of the true effect of task shifts on employment.

Results are shown in Table 7. For comparison with previous studies (e.g., Goos et al., 2014), panel A presents estimates of Eq. (5) without our main variable of interest, ∆*RTI*. Clearly, routine-task intensity is associated with employment growth for all the decades, but, consistent with the hypothesised Great Reversal (Beaudry et al., 2016), the size of this association fades over time.^22^ Panel B shows that within-occupation changes in routine task intensity have a statistically significant association with employment dynamics over the entire 30-year span of our analysis (columns 4–5)^23^ and, also, that such an effect is concentrated in the decade 1990–2000 (column 3).^24^ In this crucial transition for the US labour market, as also pointed out by Atalay et al. (2020), occupations that experienced a relatively larger decrease in routine task intensity grew faster than those with a similar level of initial routine task intensity. Importantly, the inclusion of our proxy of within-occupation task change reduces the size of the coefficient of initial routine task intensity, which becomes statistically insignificant in the last decade.

**Table 7:** Baseline estimates for employment.⁎

Panel A - Only initial RTI				
Dep: Δlog(Empl)	(1) 1980–1990	(2) 1990–2000	(3) 2000–2010	(4) 1980–2010
Initial RTI	−0.223⁎⁎⁎	−0.139⁎⁎	−0.0770⁎⁎	−0.474⁎⁎⁎
	(0.0544)	(0.0593)	(0.0387)	(0.117)
Offshorability	0.727⁎⁎	−0.411	0.0688	0.736
	(0.363)	(0.401)	(0.199)	(0.693)
Initial NRM tasks	0.274	0.0809	0.0583	0.571
	(0.183)	(0.267)	(0.0995)	(0.395)
R sq	0.317	0.179	0.380	0.365
N	29,847	28,897	28,083	26,531

Panel B - Initial and change of RTI				
Dep: Δlog(Empl)	(1) 1980–1990	(2) 1990–2000	(3) 2000–2010	(4) 1980–2010
Initial RTI	−0.216⁎⁎⁎	−0.119⁎⁎	−0.0553	−0.627⁎⁎⁎
	(0.0583)	(0.0583)	(0.0543)	(0.126)
Change in RTI	0.239	−0.237⁎⁎	0.0468	−0.426⁎⁎
	(0.241)	(0.103)	(0.0853)	(0.207)
Offshorability	0.763⁎⁎	−0.565	0.0948	0.405
	(0.364)	(0.411)	(0.206)	(0.736)
Initial NRM tasks	0.272	0.0706	0.0564	0.617
	(0.182)	(0.260)	(0.100)	(0.378)
R sq	0.319	0.187	0.381	0.370
N	29,847	28,897	28,083	26,531

Notes: OLS model. Unit of analysis: occupation, industry, year pairs. All models include industry dummies. Weights used are the start of period product of Census (1980 in column 1 and 4; 1990 in column 2; 2000 in column 3) sampling weights and annual hours of labour supply. Robust standard errors clustered at occupation level in parenthesis.

⁎ *p* < 0.1.

⁎⁎ *p* < 0.05.

⁎⁎⁎ *p* < 0.01.

The magnitude of the association between our variables of interest and employment growth is quantified in Table 8. Since both ∆*RTI* and *RTI* have no clear scale, we quantify the change in employment implied by inter-quartile changes in our variables of interest. The goal is to compare the extent to which previous estimates – based only on initial routine-task intensity – fail to account for the overall association between routine-replacing technological change and employment. As expected, differences in the initial level of *RTI* explain a larger portion of employment growth than changes in routine-task intensity. To illustrate, over the thirty years under analysis, occupations in the bottom quartile of routine task intensity grow on average 41 % more than occupations in the top quartile (row 4). In turn, the long-term inter-quartile difference in the change of routine task intensity accounts for a lower bound with a 10.8 % difference (row 5) to an upper bound with a 15.8 % difference (row 4) in employment growth. However, the magnitude of the association between task reorientation and employment growth is larger than that of the initial *RTI* in the 1990s.

**Table 8:** Quantification of employment changes.

	IQR initial RTI	IQR ΔRTI	Predicted employment change (percentage) by 1 IQR decrease of initial RTI	Predicted employment change (percentage) by 1 IQR decrease of ΔRTI
1980–1990	0.651	0.046	0.141	*−0.011*
1990–2000	0.730	0.428	0.087	0.101
2000–2010	0.939	0.420	*0.052*	*−0.020*
Long difference 1980–2010	0.797	0.354	0.408	0.158

Notes: The quantification is based on baseline results from Panel B of Table 7. Not significant effects (*p*-value < 0.1) are indicated in italics.

As discussed in Section 2, the emergence of new job titles contributes both to occupational employment growth (Lin, 2011) and to changes in occupational tasks. To account for this, we build an alternative RTI wherein we shut down the contribution of new jobs to task changes by calculating the task importance and level for all decades using decade-specific tasks and time-invariant employment weights for detailed occupational titles of the CPS 1971 Monthly File (as in Autor et al., 2003). In this way, we do not account for the emergence of ‘new jobs’ (i.e., new occupational titles not available in 1971). To account explicitly for the emergence of new occupational titles, we follow Lin (2011) and update his data to measure the cumulative number of new detailed job titles between 1980 and 2010.^25^ Our results for the RTI variable (Table 9) remain largely unchanged as regards the sign, the magnitude and the statistical significance, while the share of new job titles within the occupation is positively but not significantly correlated with employment growth.

**Table 9:** Effect on employment accounting for new jobs.⁎

Dep: Δlog(Empl), 1980–2010	RTI (benchmark)	RTI (net of contribution of new jobs)
Initial RTI	−0.595⁎⁎⁎	−0.624⁎⁎⁎
	(0.136)	(0.135)
Change in RTI	−0.441⁎⁎	−0.455⁎⁎
	(0.210)	(0.206)
Share of new jobs in occ	0.265	0.222
	(0.260)	(0.265)
Offshorability	0.323	0.385
	(0.727)	(0.719)
Initial NRM tasks	0.602	0.612
	(0.375)	(0.375)
R sq	0.371	0.371
N	26,494	26,494

Notes: OLS model. Unit of analysis: occupation, industry pairs. All models include industry dummies. Weights used are the start of period product of Census (1980) sampling weights and annual hours of labour supply. Robust standard errors clustered at occupation level in parenthesis.

⁎ *p* < 0.1.

⁎⁎ *p* < 0.05.

⁎⁎⁎ *p* < 0.01.

To summarize, explicitly accounting for within-occupation task changes does not yield different findings relative to the stylized facts established in the literature on routine-replacing technological change but uncovers novel important nuances. The association between employment and changes in the task content of occupations during the first wave of the ICT revolution in the 1990s calls for adaptation in the educational supply to fill the skill-task gap opened by the new technological regime. The subsequent decline in the importance of within-occupation task changes may indicate either successful catching-up of education with technology or a slowdown of technological change as per Beaudry et al. (2016). Our study is inconclusive in relation to these competing explanations, but points to a new, empirically testable, direction for future research.

### 4.3 Implications for wages

Task reorientation can have important implications also for wages. In a flexible labour market, such as the US, wages are usually quite elastic to demand and supply shocks. We expect that a decrease in routine task intensity increases occupational wages controlling for other intervening factors. Because workers need to learn how to perform new tasks, it may take time to observe increases in task specific productivity and, thus, rewards for task reorientation.

To isolate the association between within occupation task changes and wages it is essential to control for compositional changes that affect the mean occupational wage, in particular changes in demographic characteristics of workers. For instance, the relationship between changes in RTI and changes in wages can be ascribed to a reduction in the average experience of the occupations that de-routinize the most. Both Autor and Handel (2013) and Ross (2017) show that self-selection of workers into occupations with different degree of routine task intensity is key to estimate returns to task reorientation. In absence of a worker-level panel, we organize data in a different way. For each period, we collapse individual-level data to cells that combine workers' characteristics that are correlated with wages (occupation, industry, age, educational attainment, gender). Then, we fix the regression weights at the beginning of the period to hold workers' demographic characteristics constant. More specifically, we estimate a slightly modified version of Eq. (5):

(5) $$∆\ln\left({\mathrm{Wage}}_{\mathrm{oiage},t}\right)=\alpha +\beta_{1}\mathrm{RT}I_{o,t-1}+\beta_{2}∆\mathrm{RT}I_{o,t}+\gamma {\mathrm{NRM}}_{o,t-1}+\varphi {\mathrm{OFF}}_{o}+\delta_{t}+\delta_{i}+\varepsilon_{\mathrm{oiage},t}$$

where *Wage*_*oiage*, *t*_ is the average hourly wage of occupation *o* in industry *i*, age group *a* (16–24, 25–39, 40–54, 55–65), gender *g* (male, female) and education level *e* (less than high school, high school degree, college degree or more). Note that the coefficient measuring the association between changes in RTI and changes in wages ${\hat{\beta }}_{2}\;$is the weighted coefficient for each demographic group (Angrist and Pischke, 2008). Therefore, by holding the regression weights constant at the beginning of the period, we depurate the association between RTI and wages from the influence of compositional changes in demographic characteristics. Standard errors are clustered at the occupation level where our variables of interest are measured.

Results are shown in Table 10. In Panel A we only include the initial level of the RTI. In the long-run, we observe that both employment and wage growth are negatively correlated with the initial level of routine task intensity (column 4). In the US labour market, demand shocks, such as those driven by technological change, translate into changes of employment and wages that are aligned to each other. This holds for our favourite long-run specification but not for individual decades. While we do not observe differential wage growth related to routine task intensity over the first two decades (columns 1 and 2) a negative association exists in the last decade. In Panel B, we present a specification that includes the change in routine task intensity. Results are in line with those of Panel A. Occupations that de-routinize the most are rewarded in the long-run (column 4), but the results are driven by the last decade. Because learning new tasks takes time, a delay in wage adjustment appears a plausible explanation of this pattern. While these results are consistent with the evidence of lags in wage adjustment to job task change, a more structural approach is needed to disentangle the mechanisms that link task reorientation and wages controlling for self-selection (e.g., Autor and Handel, 2013). A study by Ross (2017) finds similar effects of the reorientation away from routine tasks on wages in a worker-level panel controlling for individual fixed effects.^26^

**Table 10:** Baseline estimates for wages.

Panel A - Only initial RTI				
Dep: Δlog(average annual wage)	(1) 1980–1990	(2) 1990–2000	(3) 2000–2010	(4) 1980–2010
Initial RTI	−0.0113	−0.00931	−0.0769⁎⁎⁎	−0.173⁎⁎⁎
	(0.0228)	(0.0134)	(0.00924)	(0.0289)
Offshorability	0.146⁎⁎	−0.0862	−0.00987	0.158⁎⁎⁎
	(0.0589)	(0.0792)	(0.0359)	(0.0577)
Initial NRM tasks	0.0393	−0.150⁎	0.00609	0.0848
	(0.0796)	(0.0836)	(0.0670)	(0.105)
R sq	0.0375	0.0480	0.0390	0.0733
N	153,792	172,077	152,197	108,921

Panel B - Initial and change of RTI				
Dep: Δlog(average annual wage)	(1) 1980–1990	(2) 1990–2000	(3)2000–2010	(4) 1980–2010
Initial RTI	−0.0117	−0.0116	−0.142⁎⁎⁎	−0.211⁎⁎⁎
	(0.0237)	(0.0130)	(0.0180)	(0.0295)
Change in RTI	−0.0158	0.0237	−0.139⁎⁎⁎	−0.111⁎⁎
	(0.0756)	(0.0214)	(0.0293)	(0.0431)
Offshorability	0.146⁎⁎	−0.0862	−0.00707	0.173⁎⁎⁎
	(0.0588)	(0.0791)	(0.0379)	(0.0553)
Initial NRM tasks	0.0364	−0.136	−0.0624	0.00293
	(0.0820)	(0.0871)	(0.0655)	(0.101)
R sq	0.0375	0.0481	0.0407	0.0742
N	153,792	172,077	152,197	108,921

Notes: OLS model. Unit of analysis: occupation, industry, gender, age (16–24, 25–39, 40–54, 55–65), education (less than high-school, high-school degree, college degree or more), year pairs. All models include industry dummies. Weights used are the start of period product of Census (1980 in column 1 and 4; 1990 in column 2; 2000 in column 3) sampling weights and annual hours of labour supply. Robust standard errors clustered at occupation level in parenthesis.

⁎ *p* < 0.1.

⁎⁎ *p* < 0.05.

⁎⁎⁎ *p* < 0.01.

Table 11 quantifies the estimated coefficients in a similar way as for employment and shows that in the long-run an interquartile decrease in routine task intensity predicts around 3.9 % of the decline of occupational wages. In turn, an interquartile decline in the initial level of RTI accounts for nearly 17 % of the decline of occupational wages. These results are broadly consistent with those on employment: within occupation task changes are important in understanding long-run labour market dynamics, but less so than the initial exposure to routine replacing technological change. In a nutshell, even a routine-intensive occupation that successfully adapts its task content can only partially escape the eventual decline of labour market opportunities.

**Table 11:** Quantification of wage changes.

	IQR initial RTI	IQR ΔRTI	Predicted wage change (percentage) by 1 IQR decrease of initial RTI	Predicted wage change (percentage) by 1 IQR decrease of ΔRTI
1980–1990	0.651	0.046	*0.008*	*0.001*
1990–2000	0.730	0.428	*0.008*	*−0.010*
2000–2010	0.939	0.420	0.133	0.058
Long difference 1980–2010	0.797	0.354	0.168	0.039

Notes: The quantification is based on baseline results from Panel B of Table 10. Not significant effects (*p*-value < 0.1) are indicated in italics.

### 4.4 Heterogeneous effects by occupational groups and macro-sectors

This final section replicates the estimation of Eq. (5) after splitting the sample by macro-occupational groups. At the cost of significantly decreasing the source of variation in the association between employment and wages growth and routinization, we seek to discern which occupations have benefited the most from changes in task content. Results in Table 12 resonate with the descriptive section, namely: within-occupation task changes are particularly important in explaining variations in employment of Clerical occupations. Notice that, as expected, reducing the source of data variation used for our estimation entails that the coefficients of both ∆*RTI* and *RTI* are less precise.

**Table 12:** Estimates of employment change by macro-occupational group.

Dep: Δlog(Empl), 1980–2010	Abstract occupations	Clerical occupations	Manual occupations	Service occupations
Initial RTI (1980)	−0.461	−1.186⁎⁎⁎	0.200	−0.463⁎
	(0.415)	(0.320)	(0.323)	(0.256)
Change in RTI (1980–2010)	0.0822	−0.751⁎⁎	0.151	−0.302
	(0.285)	(0.342)	(0.419)	(0.246)
Offshorability	1.666	−2.831⁎⁎⁎	1.293	−0.719
	(1.105)	(0.923)	(1.023)	(0.614)
Initial NRM tasks	0.522	−5.544⁎⁎⁎	1.299⁎⁎	0.721
	(0.679)	(1.399)	(0.475)	(0.767)
R sq	0.451	0.461	0.366	0.595
N	7589	6042	10,295	2480

Notes: OLS model. Unit of analysis: occupation, industry pairs. All models include industry dummies. Weights used are the start of period product of Census (1980) sampling weights and annual hours of labour supply. Robust standard errors clustered at occupation level in parenthesis.

⁎ *p* < 0.1.

⁎⁎ *p* < 0.05.

⁎⁎⁎ *p* < 0.01.

Table 13 shows the estimates of Eq. (5) after splitting our sample by macro-sectors. In both manufacturing and non-manufacturing activities, employment grow relatively faster in occupations that become relatively less-routine intensive.^27^

**Table 13:** Estimates of employment change by macro-sector.⁎⁎

Dep: Δlog(Empl), 1980–2010	Manufacturing sectors	Non-manufacturing sectors
Initial RTI (1980)	−0.840⁎⁎⁎	−0.537⁎⁎⁎
	(0.207)	(0.156)
Change in RTI (1980–2010)	−0.687⁎	−0.387⁎
	(0.369)	(0.222)
Offshorability	1.915⁎	−0.207
	(0.964)	(0.864)
Initial NRM tasks	0.911	0.454
	(0.591)	(0.423)
R sq	0.280	0.292
N	9928	16,603

Notes: OLS model. Unit of analysis: occupation, industry pairs. All models include industry dummies. Weights used are the start of period product of Census (1980) sampling weights and annual hours of labour supply. Robust standard errors clustered at occupation level in parenthesis.

⁎ *p* < 0.1.

⁎⁎ *p* < 0.05.

⁎⁎⁎ *p* < 0.01.

## 5 Concluding remarks

This paper has presented an analysis of occupational task content over the period 1980–2010 in the US. We fill a gap in the empirical literature on structural changes in labour markets by adding an important nuance to the extended literature that has primarily focused on the reallocation of workers between occupations, viz. the extensive margin. Looking at changes in job tasks within occupations, viz. the intensive margin, affords the opportunity to account for the disruptive effects of new technology beyond a short-term horizon, and to capture qualitative transformations of work activities and, thus, of the attendant skills. Moreover, analysing how the job content changes over time is relevant to identify skill gaps and to inform education and training policy. To this end, we devised a procedure to match the most comprehensive sources of occupation-specific data available for the US, DOT and O*NET, and to propose a consistent index of within-occupation routine task intensity over a thirty-year period.

Descriptive evidence based on our approach shows that within-occupation task change accounts for more than one third of the overall decline in RTI between 1980 and 2010. Beneath this aggregate pattern, however, stand important nuances. First, the within-component task change accelerates in the 1990s and decelerates in the 2000s. Second, the acceleration of the 1990s is accompanied by a divergence in the routine-intensity of jobs, with abstract occupations becoming less routine intensive. By contrast, the deceleration of the 2000s is accompanied by a substantial de-routinization of Clerical and Blue-collar occupations relative to Abstract occupations. Our exploratory regression analysis yields three main findings. First, as expected, change in computer use exhibits a positive association with within-occupation changes in analytical and interactive tasks and a negative association with changes in routine tasks. Second, changes in within-occupation RTI are associated with decennial employment growth over the entire timespan, with the strongest effect in the 1990s. Third, both employment and wages growth relatively faster in occupations that de-routinise the most over the three decades under analysis, conditional on the initial level of routine task intensity.

Overall, while we acknowledge that the exercise proposed here is but a preliminary foray into an little explored terrain, we also hope that it will inspire new research on a topic that is relevant for both scholarly and policy debates.

## CRediT authorship contribution statement

**Davide Consoli**: Conceptualization, Writing- Original draft preparation, Writing- Reviewing and Editing

**Francesco Rentocchini**: Supervision, Original draft preparation, Writing- Reviewing and Editing, Writing - Original Draft, Formal analysis, Methodology, Data

**Giovanni Marin**: Methodology, Writing- Reviewing and Editing, Formal analysis, Data

**Francesco Vona:** Supervision, Conceptualization, Writing- Original draft preparation, Writing - Original Draft, Editing, Methodology

## Declaration of competing interest

The authors declare that they have no known competing financial interests or personal relationships that could have appeared to influence the work reported in this paper.

## Data Availability

The authors do not have permission to share data.

## Appendix A Supplementary material

Supplementary material Image 1

## Footnotes

1 The occupational classification used in the two versions of DOT features about 12,000 occupations, while the occupational classification used in the two versions of O*NET that we use include, respectively, 900 (O*NET 4.0) and 924 (O*NET 18.0) occupations according to the Standard Occupational Classification (SOC) at the 8-digit level.

2 These scales are the ones used in O*NET for the sections of our interest: abilities, skills, knowledge and work activities. Other sections such as ‘work context’ are evaluated according to other specific scales (context, 1–5).

3 As much as 98 occupations (2.1 % of total employment) in O*NET 11.0 were not updated since O*NET 11.0, while the remaining 53 occupations (1.3 % of total employment) were updated compared to O*NET 4.0 by analysts and occupational experts.

4 Additional correlations and regression tables are available upon request.

5 The ‘level’ scale refers to the proficiency that is required in performing a task, while the ‘importance’ scale captures how important the task is for the occupation. Even though the two scales appear to be conceptually different, scores for the same task are always strongly correlated. The correlation between ‘level’ (0–7) and ‘importance’ (1–5) for the selected tasks is very strong: 0.93 (0.95 rank correlation) for Clerical (2.C.1.b), 0.94 (0.94 rank correlation) for Manual Dexterity (1.A.2.a.2) and 0.95 (0.95 rank correlation) for Arm-hand Steadiness (1.A.2.a.1), our proxy for non-routine manual tasks.

6 For the three tasks for which we use the level scale in O*NET, the level is >6 in just two cases: Typists (Clerical, level 6.23 for year 2010) and General office clerks (Clerical, level 6.03 for year 2010). This means that truncation at the top entails very little loss of information. Regarding truncation at the bottom, this happens in 281 cases over a total of 1932 occupation * task * year cases (14.5 %).

7 The quantile-to-quantile plot reports the quantile of the variable in the left axis (RTI in t+10 in our case) in the distribution of quantiles of the variable in the right axis (RTI in t in our case). If all dots lay on the diagonal, that means that the rank distributions of the two variables are identical. This, however, does not necessarily mean that the RTI remains constant for all occupations.

8 Computer use at work from the CPS Computer Use Supplement (October) refers to 1989 (for tasks measured in 1990), to 1997 (for tasks measured in 2000) and to 2003 (for tasks measured in 2010).

9 For descriptive purposes, we aggregate the 322 occupations into four macro-groups that roughly illustrate the main task categories under analysis: Abstract occupations for Non-Routine Cognitive tasks; Clerks for Routine Cognitive; Blue Collar for Routine Manual; Service jobs for Non-Routine Manual. The aggregation of occupations into the four macro-groups is defined in Acemoglu and Autor (2011) while the correspondence between *OCC1990* occupations and macro-groups is based on Dorn (2009). See Table A2 in the Appendix A2.

10 A preliminary sketch about the relevance of within-occupation task changes can be grasped by looking at decade-to-decade transition tables (Table B1 in Appendix B). When breaking the RTI index into quintiles, a large number of occupations lie outside the main diagonal of the transition matrix (18 % for 1980–1990, 56 % for 1990–2000, 48 % for 2000–2010). This also implies that we have sufficient data variation to distinguish between the influence of the initial RTI and that of within-occupation task changes on employment dynamics.

11 In principle, the two between components can be further decomposed to inspect the possible covariance between changes in industry and occupational structure and levels and changes in RTI. This full decomposition will read as:

$$∆\mathrm{RTI}=\sum_{i,o}\left[\bar{\delta_{i}\phi_{i,o}}∆\mathrm{RT}I_{o}+\bar{\delta_{i}}∆\phi_{i,o}\mathrm{RT}I_{o}^{1980}+∆\delta_{i}\bar{\phi_{\mathrm{io}}}\mathrm{RT}I_{o}^{1980}+\bar{\delta_{i}}∆\phi_{i,o}\left(\bar{\mathrm{RT}I_{o}}-\mathrm{RT}I_{o}^{1980}\right)+∆\delta_{i}\bar{\phi_{\mathrm{io}}}\left(\bar{\mathrm{RT}I_{o}}-\mathrm{RT}I_{o}^{1980}\right)\right]$$

where $\bar{\delta_{i}}\text{∆}\phi_{i,o}\mathrm{RT}I_{o}^{1980}$ and $\text{∆}\delta_{i}\bar{\phi_{\mathrm{io}}}\mathrm{RT}I_{o}^{1980}$ are, respectively, the pure between-occupation and between-industry components (calculated with the initial RTI). On the other hand, $\bar{\delta_{i}}\text{∆}\phi_{i,o}\left(\bar{\mathrm{RT}I_{o}}-\mathrm{RT}I_{o}^{1980}\right)$ and $\text{∆}\delta_{i}\bar{\phi_{\mathrm{io}}}\left(\bar{\mathrm{RT}I_{o}}-\mathrm{RT}I_{o}^{1980}\right)$ represent the covariance components of, respectively, the between-occupation and between-industry components. However, the two covariance terms $\bar{\delta_{i}}\text{∆}\phi_{i,o}\left(\bar{\mathrm{RT}I_{o}}-\mathrm{RT}I_{o}^{1980}\right)$ and $\text{∆}\delta_{i}\bar{\phi_{\mathrm{io}}}\left(\bar{\mathrm{RT}I_{o}}-\mathrm{RT}I_{o}^{1980}\right)$ appear relatively small compared to the other terms, so we focus on the simpler decomposition. Details on the results of the full decomposition are available in Tables B2, B3 and B4 of the Appendix B.

12 The change in the task content of an occupation is bounded in the sense that a high-skilled job at the top of the mathematical score (i.e. financial quantitative analyst) cannot go above that level even if technology becomes more complex and, thus, requires an even higher level of proficiency in coding (i.e. for algorithmic trading). This limits the possibility to observe the emergence of new tasks in top occupations, especially once that occupation has reached the bottom of the RTI index, which is more likely to occur in a later decade than in earlier ones. Thus, there are higher changes to underestimate within-occupation task chances in the last decade than in the first ones. Note that we use O*NET version 5.0 (April 2003) for the 2000s decade. This can be also due to O*NET being more conservative in updating the task description and that the 2000–2010 variation is de facto a variation between 2003 and 2010 since the early versions of O*NET cannot be used due to lack of standardisation in the data collection methodology.

13 Notice that considering only the between-occupations component will underestimate the shift away from routine tasks. Moreover, the between-occupations component varies less across decades.

14 This panel combines the dimensions from the two previous panels into one. In this case, a de-routinisation process within an occupation becomes, as expected, positively correlated with employment growth conditional on the level of initial RTI. This is later confirmed by the results we obtained in the regression analysis, with the added value of being robust after controlling for additional covariates.

15 Because our measure of RTI is bounded, occupations in the first quantile of routine task intensity cannot be among the first two quintiles of de-routinization.

16 More specifically, information on computer use at work is available for CPS Computer Use Supplement (October) for years 1989, 1993, 1997, 2001 and 2003.

17 We measure computer use as the share of workers that use a computer at work in an occupation. We use 1997 as a proxy for computer use in 2000 as we expect computer use to affect tasks with a lag. However, results are qualitatively unaffected if we use computer use in 2003 to proxy the 2000 values.

18 The purpose of this regression model is also to provide a further check for our measure of within-occupation task shifts, particularly with respect to the role of computer adoption in the workplace. We included the initial task share in the regression because we are examining a natural process of catching-up and, as in all dynamic processes, controlling for initial conditions is extremely relevant. We want to see whether after controlling for differences across occupations in their initial level of routinisation, technological change contributes to the change in routinisation within time. Our results are also robust to dropping the initial level of task intensity (available from the authors upon request).

19 We estimate the association between routine task intensity and employment growth building upon the approach from the existing empirical literature (see for example Goos et al., 2014 and Gregory, Salomons, Zierahn 2019). We control for the initial level of routine task intensity and add the change in routine task intensity within occupation as our main variable of interest.

20 Offshorability is included as a control to capture occupation-level exposure to activities that are potentially offshorable. Recent evidence shows that the remarkable pace of expansion of China and of various emerging economies had a negative employment effect (Pierce and Schott, 2012; Autor et al., 2016) due to the impact of greater fragmentation of supply chains (Baldwin, 2011) on the offshoring of routine tasks involving minimal complexity (Blinderm, 2009). It is worth noticing that the China's shock is also controlled for by including industry level dummies. We also control for the non-routine manual intensity as occupations that are intensive in non-routine manual tasks tend to absorb the routine workers displaced by technological change. Note that the work from ALM (2003) suggests a limited effect of routine-replacing technological change on the labour market outcomes of occupations intensive in non-routine manual tasks. However, later research contends that the demand for such occupations increases due to two main effects: i) occupations that are intensive in non-routine manual tasks absorb workers displaced by technological change, and ii) non-routine manual tasks are difficult to substitute with computers (Autor et al., 2006; Autor and Dorn, 2013; Goos and Manning, 2007).

21 In the stacked model we slightly amend the specification of equation 5 by interacting industry dummies and offshorability, which is time-invariant, with time dummies.

22 The number of observations is different in each decade because the panel (occupation-by-industry-by-year) is not balanced. This happens because a number of occupation-industry cells might have zero employment either in the first year of the decade, or in the last year or both. However, unbalanced cells are <2 % of initial or final total employment.

23 When employing a fixed effect estimator instead of a first difference one in a way similar to previous work (Goos et al., 2014; Gregory et al., 2019), a coefficient of similar magnitude to the stacked regression is obtained. Due to the higher extent of serial correlation in the error terms and the higher efficiency of first difference estimator to this respect, we provide results in first difference for the remaining of the paper.

24 Tables C1, C2 and C3 in Appendix C show that the results are robust using the time-invariant weights from the April 1971 CPS Monthly File, including non-routine manual tasks in the RTI index and using a more parsimonious specification without the task measures for offshorability and for the importance of non-routine manual tasks.

25 Data on new jobs built by Lin (2011) are available at https://github.com/jeffrlin/new-work while the full list of detailed Census occupational titles for 2010 are available at https://www2.census.gov/programs-surveys/demo/guidance/industry-occupation/2010-occupation-index-08132013.xls.

26 Similarly to our employment results, controlling for new jobs (first column) and shutting down the contribution of new jobs to within-occupation RTI change (second column) has no influence on our results (Table C5 in Appendix C), which remain robust and in line with the ones of Table 10 in terms of sign, magnitude and statistical significance.

27 Table C6 and C7 in the appendix contain a similar exercise about the heterogenous effects of occupational groups and macro-sectors on wages.
