# Supplementary material for: Routinization, within-occupation task changes and long-run employment dynamics
Source: Res Policy. 2023 Jan;52(1):104658. doi: 10.1016/j.respol.2022.104658 (PMC9746329; doi:10.1016/j.respol.2022.104658)
Supplement: Supplementary file 1 — Supplementary material [file mmc1.docx]

# Appendix A – Additional evidence on task measures

## A2 Definition of macro-occupational groups

Table A2 – Definition of macro occupational groups (based on Dorn, 1999, and Acemoglu and Autor, 2011)

| OCC1990 | Occupation name |
| --- | --- |
| Abstract occupations | |
| 4 | Chief executives, public administrators, and legislators |
| 7 | Financial managers |
| 8 | Human resources and labor relations managers |
| 13 | Managers and specialists in marketing, advert., PR |
| 14 | Managers in education and related fields |
| 15 | Managers of medicine and health occupations |
| 18 | Managers of properties and real estate |
| 22 | Managers and administrators, n.e.c. |
| 23 | Accountants and auditors |
| 24 | Insurance underwriters |
| 25 | Other financial specialists |
| 26 | Management analysts |
| 27 | Personnel, HR, training, and labor rel. specialists |
| 28 | Purchasing agents and buyers of farm products |
| 29 | Buyers, wholesale and retail trade |
| 33 | Purchasing managers, agents, and buyers, n.e.c. |
| 34 | Business and promotion agents |
| 36 | Inspectors and compliance officers, outside |
| 37 | Management support occupations |
| 43 | Architects |
| 44 | Aerospace engineers |
| 45 | Metallurgical and materials engineers |
| 47 | Petroleum, mining, and geological engineers |
| 48 | Chemical engineers |
| 53 | Civil engineers |
| 55 | Electrical engineers |
| 56 | Industrial engineers |
| 57 | Mechanical engineers |
| 59 | Engineers and other professionals, n.e.c. |
| 64 | Computer systems analysts and computer scientists |
| 65 | Operations and systems researchers and analysts |
| 66 | Actuaries |
| 68 | Mathematicians and statisticians |
| 69 | Physicists and astronomists |
| 73 | Chemists |
| 74 | Atmospheric and space scientists |
| 75 | Geologists |
| 76 | Physical scientists, n.e.c. |
| 77 | Agricultural and food scientists |
| 78 | Biological scientists |
| 79 | Foresters and conservation scientists |
| 83 | Medical scientists |
| 84 | Physicians |
| 85 | Dentists |
| 86 | Veterinarians |
| 87 | Optometrists |
| 88 | Podiatrists |
| 89 | Other health and therapy occupations |
| 95 | Registered nurses |
| 96 | Pharmacists |
| 97 | Dieticians and nutritionists |
| 98 | Respiratory therapists |
| 99 | Occupational therapists |
| 103 | Physical therapists |
| 104 | Speech therapists |
| 105 | Therapists, n.e.c. |
| 106 | Physicians' assistants |
| 154 | Subject instructors, college |
| 155 | Kindergarten and earlier school teachers |
| 156 | Primary school teachers |
| 157 | Secondary school teachers |
| 158 | Special education teachers |
| 159 | Teachers, n.e.c. |
| 164 | Librarians |
| 165 | Archivists and curators |
| 166 | Economists, market and survey researchers |
| 167 | Psychologists |
| 169 | Social scientists and sociologists, n.e.c. |
| 173 | Urban and regional planners |
| 178 | Lawyers and judges |
| 183 | Writers and authors |
| 184 | Technical writers |
| 185 | Designers |
| 186 | Musicians and composers |
| 187 | Actors, directors, and producers |
| 188 | Painters, sculptors, craft-artists, and print-makers |
| 189 | Photographers |
| 193 | Dancers |
| 194 | Art/entertainment performers and related occs |
| 195 | Editors and reporters |
| 198 | Announcers |
| 199 | Athletes, sports instructors, and officials |
| 203 | Clinical laboratory technologies and technicians |
| 204 | Dental hygienists |
| 205 | Health record technologists and technicians |
| 206 | Radiologic technologists and technicians |
| 207 | Licensed practical nurses |
| 208 | Health technologists and technicians, n.e.c. |
| 214 | Engineering technicians |
| 217 | Drafters |
| 218 | Surveryors, cartographers, mapping scientists/techs |
| 223 | Biological technicians |
| 224 | Chemical technicians |
| 225 | Other science technicians |
| 226 | Airplane pilots and navigators |
| 227 | Air traffic controllers |
| 228 | Broadcast equipment operators |
| 229 | Computer software developers |
| 234 | Legal assistants and paralegals |
| 473 | Farmers (owners and tenants) |
| 475 | Farm managers |
| 677 | Optical goods workers |
| 678 | Dental laboratory and medical applicance technicians |
| Blue-collar occupations | |
| 35 | Construction inspectors |
| 233 | Programmers of numerically controlled machine tools |
| 408 | Laundry and dry cleaning workers |
| 503 | Supervisors of mechanics and repairers |
| 505 | Automobile mechanics and repairers |
| 507 | Bus, truck, and stationary engine mechanics |
| 508 | Aircraft mechanics |
| 509 | Small engine repairers |
| 514 | Auto body repairers |
| 516 | Heavy equipement and farm equipment mechanics |
| 518 | Industrial machinery repairers |
| 519 | Machinery maintenance occupations |
| 523 | Repairers of industrial electrical equipment |
| 525 | Repairers of data processing equipment |
| 526 | Repairers of household appliances and power tools |
| 527 | Telecom and line installers and repairers |
| 533 | Repairers of electrical equipment, n.e.c. |
| 534 | Heating, air conditioning, and refrigeration mechanics |
| 535 | Precision makers, repairers, and smiths |
| 536 | Locksmiths and safe repairers |
| 539 | Repairers of mechanical controls and valves |
| 543 | Elevator installers and repairers |
| 544 | Millwrights |
| 549 | Mechanics and repairers, n.e.c. |
| 558 | Supervisors of construction work |
| 563 | Masons, tilers, and carpet installers |
| 567 | Carpenters |
| 573 | Drywall installers |
| 575 | Electricians |
| 577 | Electric power installers and repairers |
| 579 | Painters, construction and maintenance |
| 583 | Paperhangers |
| 584 | Plasterers |
| 585 | Plumbers, pipe fitters, and steamfitters |
| 588 | Concrete and cement workers |
| 589 | Glaziers |
| 593 | Insulation workers |
| 594 | Paving, surfacing, and tamping equipment operators |
| 595 | Roofers and slaters |
| 597 | Structural metal workers |
| 598 | Drillers of earth |
| 599 | Misc. construction and related occupations |
| 614 | Drillers of oil wells |
| 615 | Explosives workers |
| 616 | Miners |
| 617 | Other mining occupations |
| 628 | Production supervisors or foremen |
| 634 | Tool and die makers and die setters |
| 637 | Machinists |
| 643 | Boilermakers |
| 644 | Precision grinders and fitters |
| 645 | Patternmakers and model makers |
| 649 | Engravers |
| 657 | Cabinetmakers and bench carpeters |
| 658 | Furniture/wood finishers, other prec. wood workers |
| 666 | Dressmakers, seamstresses, and tailors |
| 668 | Upholsterers |
| 669 | Shoemakers, other prec. apparel and fabric workers |
| 675 | Hand molders and shapers, except jewelers |
| 679 | Bookbinders |
| 686 | Butchers and meat cutters |
| 687 | Bakers |
| 688 | Batch food makers |
| 694 | Water and sewage treatment plant operators |
| 695 | Power plant operators |
| 696 | Plant and system operators, stationary engineers |
| 699 | Other plant and system operators |
| 703 | Lathe, milling, and turning machine operatives |
| 706 | Punching and stamping press operatives |
| 707 | Rollers, roll hands, and finishers of metal |
| 708 | Drilling and boring machine operators |
| 709 | Grinding, abrading, buffing, and polishing workers |
| 713 | Forge and hammer operators |
| 719 | Molders and casting machine operators |
| 723 | Metal platers |
| 724 | Heat treating equipment operators |
| 727 | Sawing machine operators and sawyers |
| 729 | Nail, tacking, shaping and joining mach ops (wood) |
| 733 | Other woodworking machine operators |
| 734 | Printing machine operators, n.e.c. |
| 736 | Typesetters and compositors |
| 738 | W inding and twisting textile and apparel operatives |
| 739 | Knitters, loopers, and toppers textile operatives |
| 743 | Textile cutting and dyeing machine operators |
| 744 | Textile sewing machine operators |
| 745 | Shoemaking machine operators |
| 747 | Clothing pressing machine operators |
| 749 | Miscellanious textile machine operators |
| 753 | Cementing and gluing machne operators |
| 754 | Packers, fillers, and wrappers |
| 755 | Extruding and forming machine operators |
| 756 | Mixing and blending machine operators |
| 757 | Separating, filtering, and clarifying machine operators |
| 763 | Food roasting and baking machine operators |
| 764 | Washing, cleaning, and pickling machine operators |
| 765 | Paper folding machine operators |
| 766 | Furnance, kiln, and oven operators, apart from food |
| 769 | Slicing, cutting, crushing and grinding machine |
| 774 | Photographic process workers |
| 779 | Machine operators, n.e.c. |
| 783 | Welders, solderers, and metal cutters |
| 785 | Assemblers of electrical equipment |
| 799 | Production checkers, graders, and sorters in |
| 803 | Supervisors of motor vehicle transportation |
| 804 | Truck, delivery, and tractor drivers |
| 808 | Bus drivers |
| 809 | Taxi cab drivers and chauffeurs |
| 813 | Parking lot attendants |
| 823 | Railroad conductors and yardmasters |
| 824 | Locomotive operators: engineers and firemen |
| 825 | Railroad brake, coupler, and switch operators |
| 829 | Ship crews and marine engineers |
| 844 | Operating engineers of construction equipment |
| 848 | Crane, derrick, winch, hoist, longshore operators |
| 853 | Excavating and loading machine operators |
| 859 | Stevedores and misc. material moving occupations |
| 865 | Helpers, constructions |
| 866 | Helpers, surveyors |
| 869 | Construction laborers |
| 873 | Production helpers |
| 875 | Garbage and recyclable material collectors |
| 878 | Machine feeders and offbearers |
| 885 | Garage and service station related occupations |
| 887 | Vehicle washers and equipment cleaners |
| 888 | Packers and packagers by hand |
| 889 | Laborers, freight, stock, and material handlers, n.e.c. |
| Clerical occupations | |
| 243 | Sales supervisors and proprietors |
| 253 | Insurance sales occupations |
| 254 | Real estate sales occupations |
| 255 | Financial service sales occupations |
| 256 | Advertising and related sales jobs |
| 258 | Sales engineers |
| 275 | Retail salespersons and sales clerks |
| 276 | Cashiers |
| 277 | Door-to-door sales, street sales, and news vendors |
| 283 | Sales demonstrators, promoters, and models |
| 303 | Office supervisors |
| 308 | Computer and peripheral equipment operators |
| 313 | Secretaries and stenographers |
| 315 | Typists |
| 316 | Interviewers, enumerators, and surveyors |
| 317 | Hotel clerks |
| 318 | Transportation ticket and reservation agents |
| 319 | Receptionists and other information clerks |
| 326 | Correspondence and order clerks |
| 328 | Human resources clerks, excl payroll and timekeeping |
| 329 | Library assistants |
| 335 | File clerks |
| 336 | Records clerks |
| 337 | Bookkeepers and accounting and auditing clerks |
| 338 | Payroll and timekeeping clerks |
| 344 | Billing clerks and related financial records processing |
| 346 | Mail and paper handlers |
| 347 | Office machine operators, n.e.c. |
| 348 | Telephone operators |
| 349 | Other telecom operators |
| 354 | Postal clerks, exluding mail carriers |
| 355 | Mail carriers for postal service |
| 356 | Mail clerks, outside of post office |
| 357 | Messengers |
| 359 | Dispatchers |
| 364 | Shipping and receiving clerks |
| 365 | Stock and inventory clerks |
| 366 | Meter readers |
| 368 | Weighers, measurers, and checkers |
| 373 | Material recording, sched., prod., plan., expediting cl. |
| 375 | Insurance adjusters, examiners, and investigators |
| 376 | Customer service reps, invest., adjusters, excl. insur. |
| 377 | Eligibility clerks for government prog., social welfare |
| 378 | Bill and account collectors |
| 379 | General office clerks |
| 383 | Bank tellers |
| 384 | Proofreaders |
| 385 | Data entry keyers |
| 386 | Statistical clerks |
| 389 | Administrative support jobs, n.e.c. |
| Service occupations | |
| 19 | Funeral directors |
| 163 | Vocational and educational counselors |
| 174 | Social workers |
| 176 | Clergy and religious workers |
| 177 | Welfare service workers |
| 405 | Housekeepers, maids, butlers, and cleaners |
| 415 | Supervisors of guards |
| 417 | Fire fighting, fire prevention, and fire inspection occs |
| 418 | Police and detectives, public service |
| 423 | Sheriffs, bailiffs, correctional institution officers |
| 425 | Crossing guards |
| 426 | Guards and police, except public service |
| 427 | Protective service, n.e.c. |
| 434 | Bartenders |
| 435 | Waiters and waitresses |
| 436 | Cooks |
| 439 | Food preparation workers |
| 444 | Miscellanious food preparation and service workers |
| 445 | Dental Assistants |
| 447 | Health and nursing aides |
| 448 | Supervisors of cleaning and building service |
| 450 | Superv. of landscaping, lawn service, groundskeeping |
| 451 | Gardeners and groundskeepers |
| 453 | Janitors |
| 455 | Pest control occupations |
| 457 | Barbers |
| 458 | Hairdressers and cosmetologists |
| 459 | Recreation facility attendants |
| 461 | Guides |
| 462 | Ushers |
| 464 | Baggage porters, bellhops and concierges |
| 466 | Recreation and fitness workers |
| 467 | Motion picture projectionists |
| 468 | Child care workers |
| 469 | Personal service occupations, n.e.c |
| 470 | Supervisors of personal service jobs, n.e.c |
| 471 | Public transportation attendants and inspectors |
| 472 | Animal caretakers, except farm |
| Farm occupations | |
| 479 | Farm workers, incl. nursery farming |
| 488 | Graders and sorters of agricultural products |
| 489 | Inspectors of agricultural products |
| 496 | Timber, logging, and forestry workers |
| 498 | Fishers, marine life cultivators, hunters, and kindred |

## A3 Quantile-to-quantile plots for selected tasks

The first validation of our matching procedure compares the distribution of task-related variables across different decades. We evaluate quantile-to-quantile plots which report the quantile of the variable in the left axis (in t+10 in our case) within the distribution of quantiles of the variable in the right axis (in t). When all dots lie on the diagonal, the rank distribution of the two variables is identical. A constant rank distribution does not necessarily mean a constant task across years. Results for all decades are reported in Figures A1, A2, A3, A4 and A5.

We do not observe any systematic differences in average tasks between years 1990 and 2000 (when O*NET was first introduced), nor in previous or subsequent periods (1980-1990 and 2000-2010). This is to say that, if systematic differences in the value of our task measures exist when matching DOT and O*NET, they are not necessarily due to our matching procedure. Even when some differences are apparent (e.g., Cleric and Manual in Figures A3 and A4), these differences cancel each other out when we aggregate information for the four task measures into our routinisation index (Figure A5).

Figure A1 – Quantile-to-quantile plot for MATH


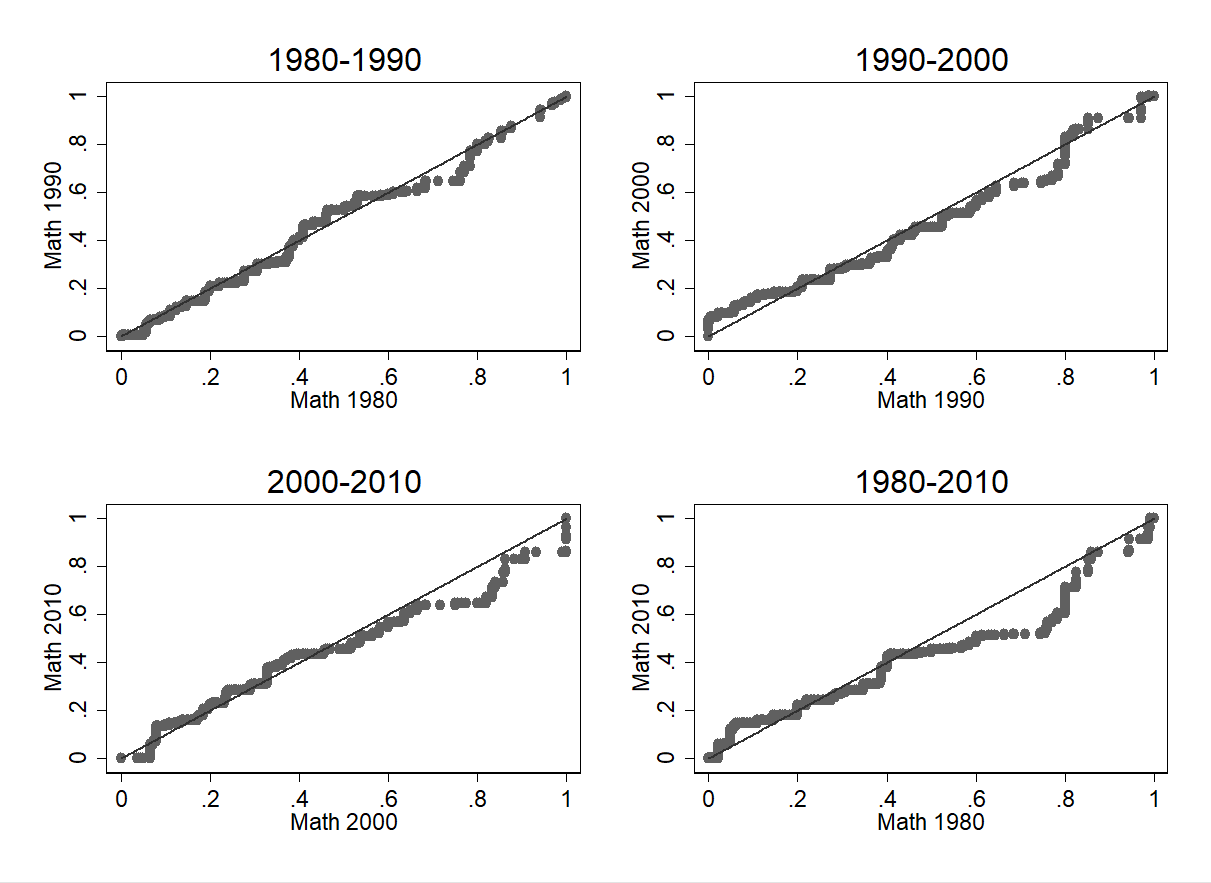


Notes: Quantiles weighted with the start of period product of Census (1980; 1990; 2000) sampling weights and annual hours of labour supply.

Figure A2 – Quantile-to-quantile plot for LANGUAGE


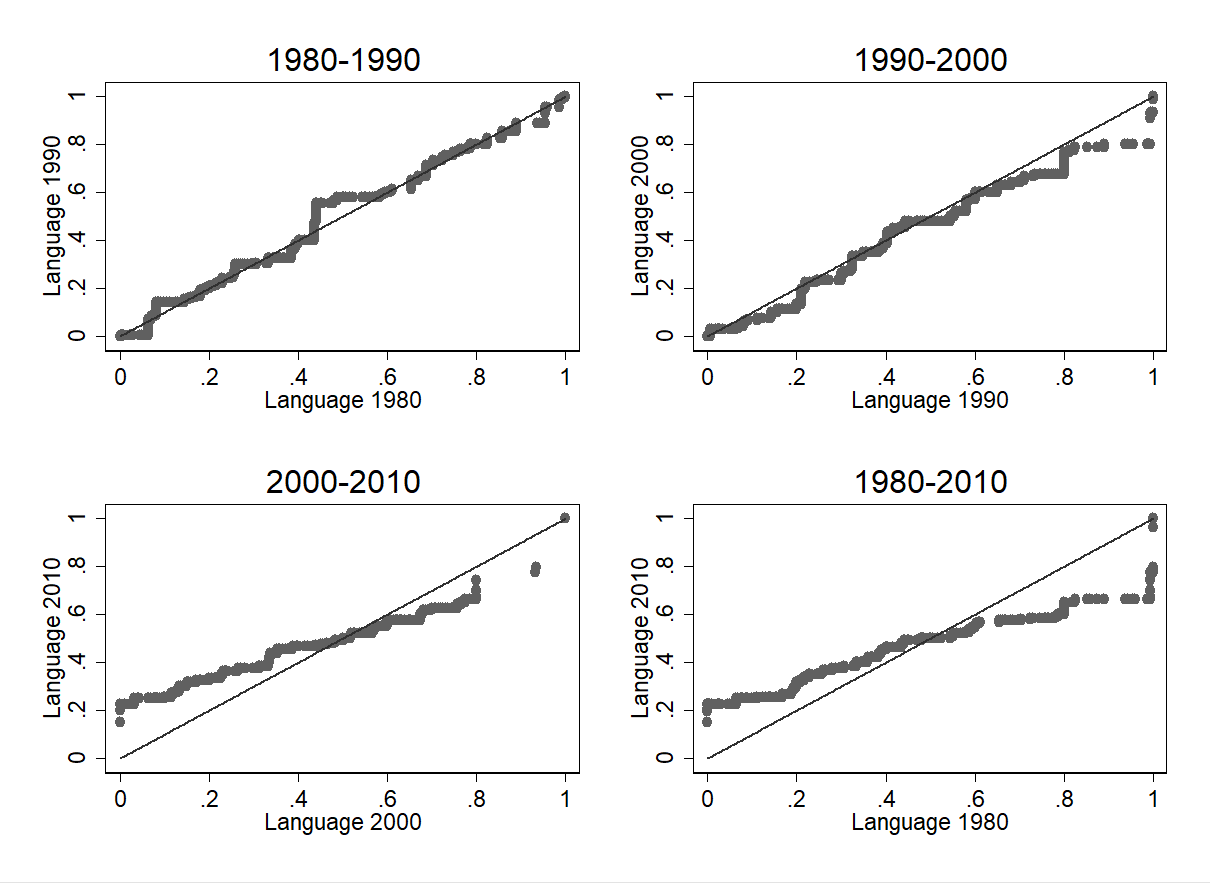


Notes: Quantiles weighted with the start of period product of Census (1980; 1990; 2000) sampling weights and annual hours of labour supply.

Figure A3 – Quantile-to-quantile plot for CLERIC


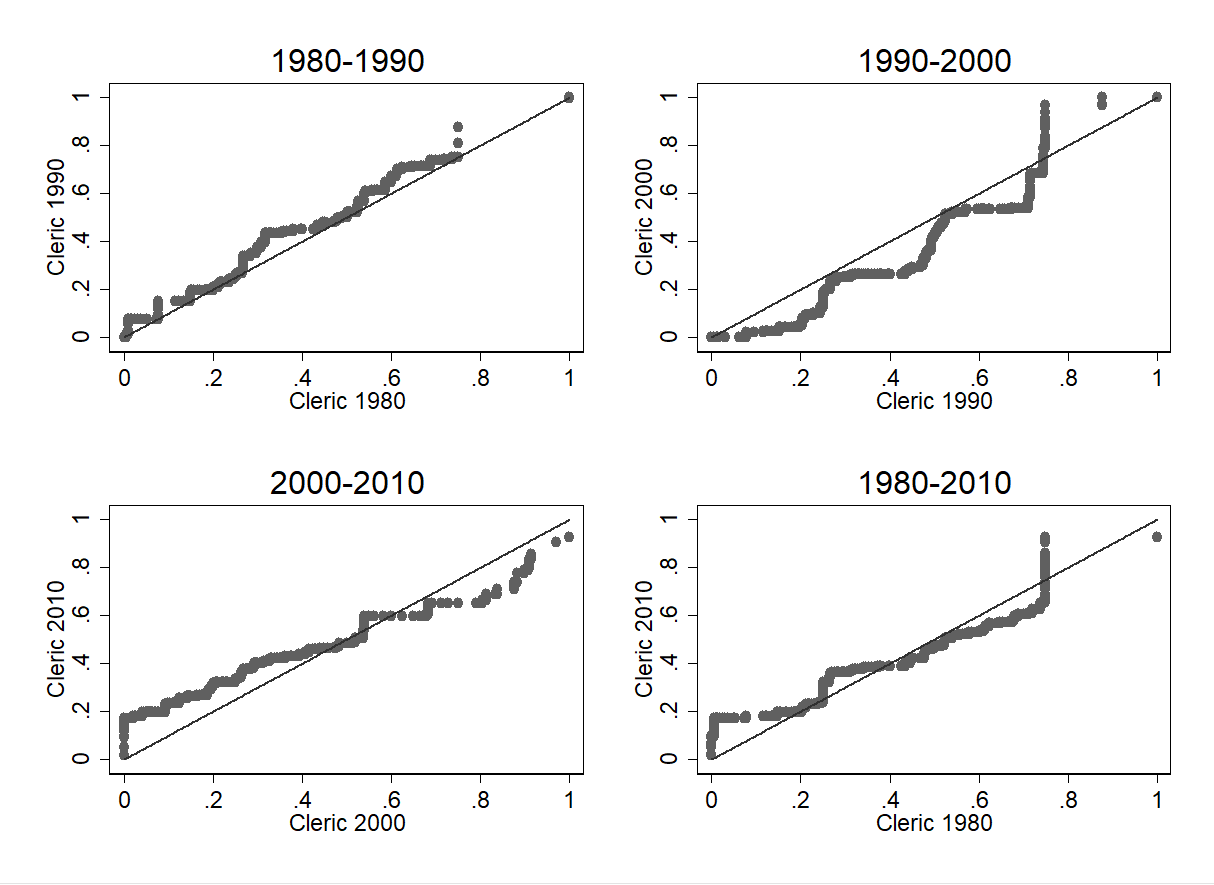


Notes: Quantiles weighted with the start of period product of Census (1980; 1990; 2000) sampling weights and annual hours of labour supply.

Figure A4 – Quantile-to-quantile plot for MANUAL


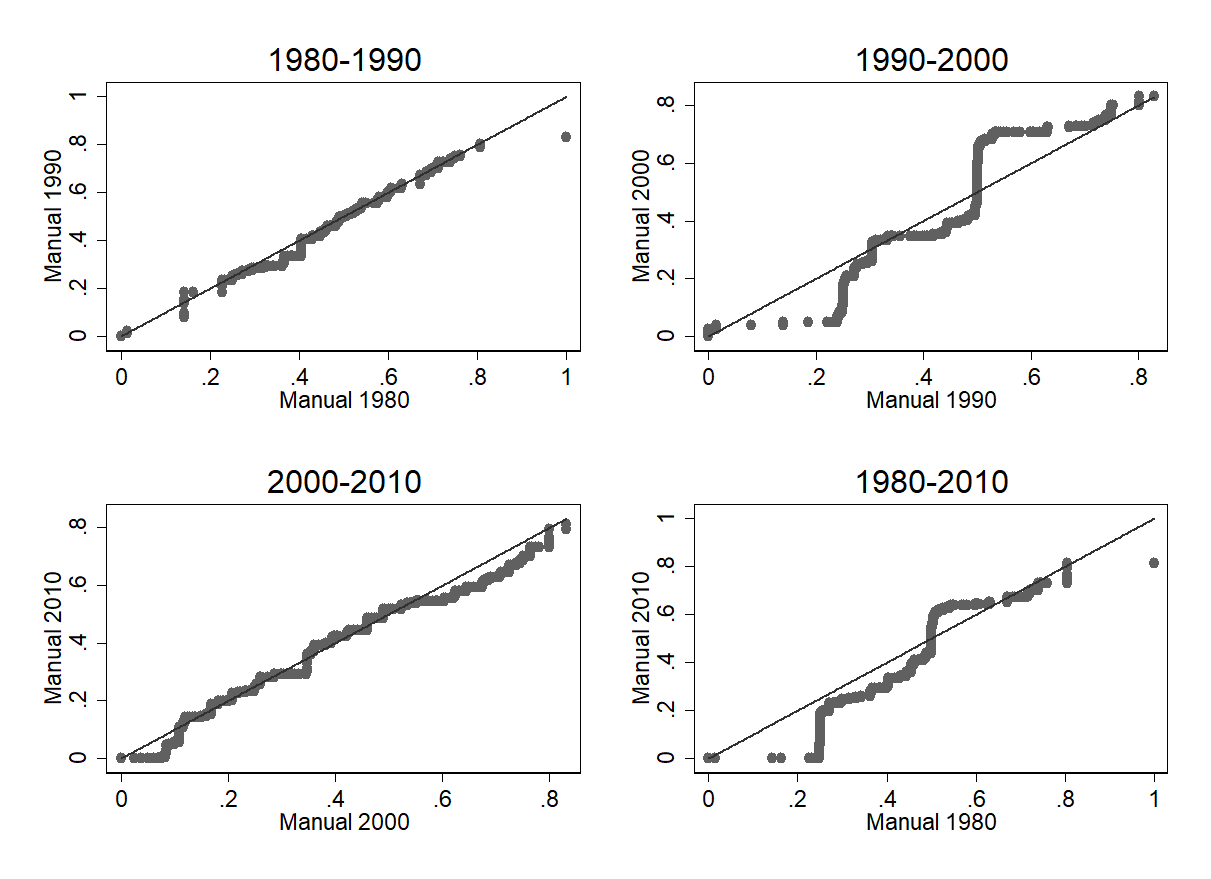


Notes: Quantiles weighted with the start of period product of Census (1980; 1990; 2000) sampling weights and annual hours of labour supply.

Figure A5 – Quantile-to-quantile plot for RTI


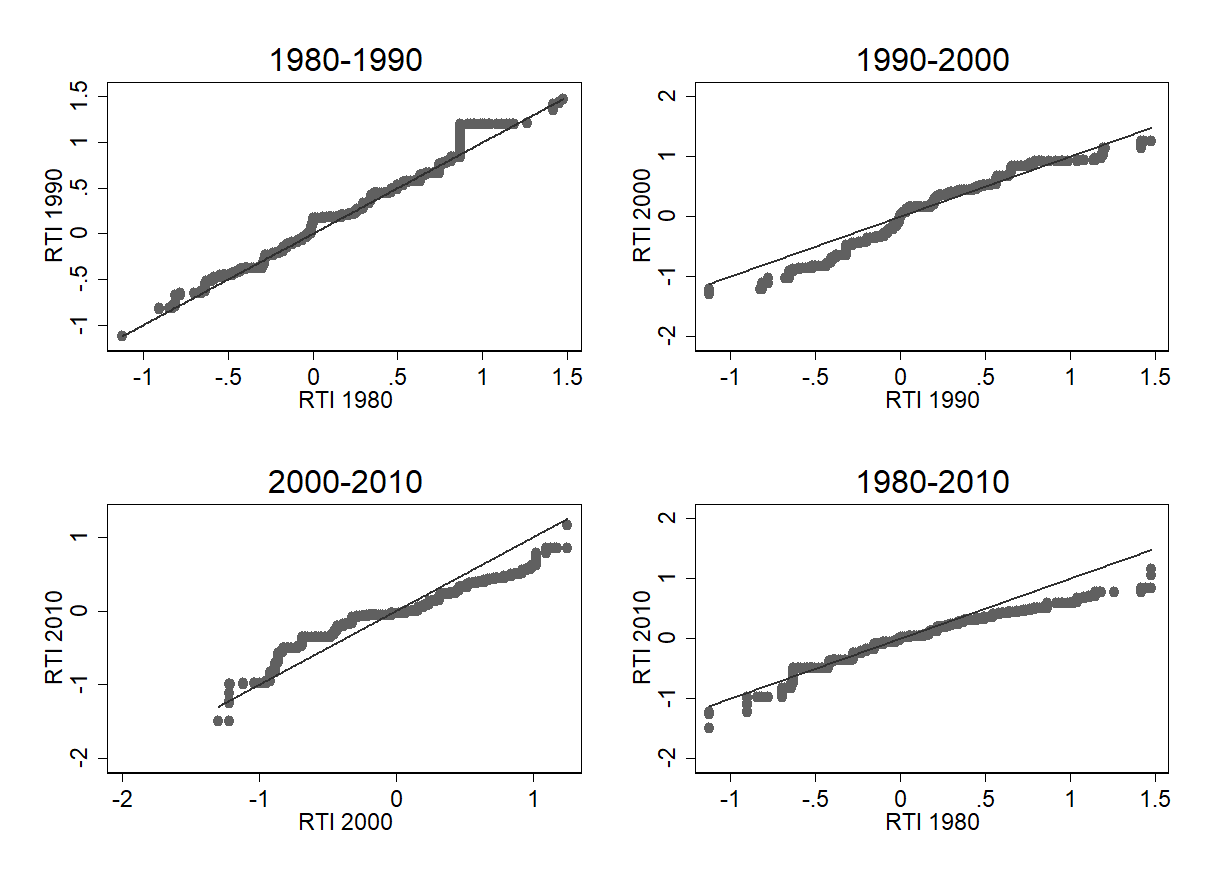


Notes: Quantiles weighted with the start of period product of Census (1980; 1990; 2000) sampling weights and annual hours of labour supply.

## A4 Test of the difference in moments of tasks between decades

To better analyse our matching between task measures in DOT and O*NET we test for differences in the moments (from first to fourth) of the empirical distribution of tasks across occupations over different decades. We employ bootstrap tests (500 repetitions) for all moments. Results are reported in Table A2.

Table A3 – Bootstrap tests for the moments of DOT and O*NET distributions

| 1990-1980 (DOT91 - DOT77) | | | | | | |
| --- | --- | --- | --- | --- | --- | --- |
|  | Math | Language | Cleric | Manual | RTI | |
| Difference in average | 0.014 | 0.025 | 0.036 | 0.036 | -0.022 | |
|  | (0.035) | (0.040) | (0.034) | (0.034) | (0.075) | |
| Difference in standard deviation | 0.008 | 0.008 | -0.001 | -0.001 | 0.017 | |
|  | (0.017) | (0.019) | (0.020) | (0.020) | (0.046) | |
| Difference in skewness | -0.157 | -0.147 | -0.172 | -0.172 | 0.172 | |
|  | (0.285) | (0.305) | (0.227) | (0.227) | (0.287) | |
| Difference in kurtosis | -0.253 | -0.170 | 0.0316 | 0.0316 | 0.0312 | |
|  | (0.548) | (0.430) | (0.318) | (0.318) | (0.556) | |
| 2000-1990 (O*NET2000-DOT91) | | | | | | |
|  | Math | Language | Cleric | Manual | RTI | |
| Difference in average | -0.010 | -0.021 | -0.070** | -0.024 | -0.107 | |
|  | (0.031) | (0.037) | (0.033) | (0.026) | (0.0838) | |
| Difference in standard deviation | -0.029* | -0.012 | 0.045** | 0.070*** | 0.114** | |
|  | (0.016) | (0.017) | (0.023) | (0.011) | (0.0448) | |
| Difference in skewness | 0.418 | -0.203 | 0.637*** | -0.089 | -0.521* | |
|  | (0.305) | (0.287) | (0.212) | (0.256) | (0.272) | |
| Difference in kurtosis | 0.854 | -0.149 | 0.713* | -0.744*** | -0.883** | |
|  | (0.653) | (0.310) | (0.396) | (0.271) | (0.389) | |
| 2010-2000 (O*NET2010-O*NET2000) | | | | | | |
|  | Math | Language | Cleric | Manual | RTI | |
| Difference in average | -0.008 | 0.049* | 0.045 | -0.021 | -0.041 | |
|  | (0.026) | (0.028) | (0.029) | (0.029) | (0.076) | |
| Difference in standard deviation | -0.019 | -0.096*** | -0.079*** | -0.010 | -0.189*** | |
|  | (0.016) | (0.013) | (0.027) | (0.013) | (0.035) | |
| Difference in skewness | -0.257 | 0.143 | 0.067 | -0.264 | -0.132 | |
|  | (0.349) | (0.305) | (0.254) | (0.245) | (0.279) | |
| Difference in kurtosis | 0.312 | 1.091 | 0.688 | 0.012 | 0.908* | |
|  | (0.896) | (0.690) | (0.567) | (0.234) | (0.540) | |
| Notes: 500 repetitions on random bootstrap samples. Standard deviation of the test is reported in parenthesis. * p<0.1, ** p<0.05, *** p<0.01. Notes: Weights are the product of Census (1980; 1990; 2000) sampling weights and annual hours of labour supply. | | | | | |  |

First, we do not find any significant differences in the moments of the distributions of all tasks between 1980 and 1990 (i.e., within DOT). Second, we do observe some significant differences in the moments of the distributions between 1990 and 2000 for routine tasks (Cleric and Manual) and for the RTI, while no difference is found for non-routine tasks (Math and Language). These differences are, however, generally small in magnitude. Finally, significant differences (again relatively small) are also found between 2000 and 2010, i.e., within the O*NET era, suggesting that the 1990-2000 change may not be due primarily to the systematic differences between DOT and O*NET, which would have suggested that our match was not effective.

## A5 Validation of task measures based on occupational computer use at work

Finally, as an additional robustness check we consider the relationship between on-the-job computer use of occupations (from different CPS Computer Use Supplement of October) and task measures (component-by-component and RTI). Results are reported in Table A3. All relationships are found to be strongly statistically significant and to have the expected sign: positively correlated with abstract tasks (MATH and LANGUAGE) and routine cognitive tasks (CLERIC), negative correlated with manual tasks (routine and non-routine manual tasks) and negatively correlated with the RTI. These strong correlations are consistent across different decades, suggesting that computer use is correlated with occupational task intensity with the expected sign both within DOT and within O*NET.

Table A4 –Task measures and computer use: levels of tasks

|  | (1) | (2) | (3) | (4) | (5) | (6) |
| --- | --- | --- | --- | --- | --- | --- |
|  | Math | Language | Cleric | Manual | RTI | NRM |
| Levels in 1990 (DOT) | | | | | | |
| Computer use in 1989 | 0.611*** | 0.765*** | 0.665*** | -0.276*** | -1.176*** | -0.295*** |
|  | (0.082) | (0.064) | (0.056) | (0.058) | (0.188) | (0.056) |
| R squared | 0.403 | 0.503 | 0.552 | 0.216 | 0.369 | 0.234 |
| Levels in 2000 (O*NET) | | | | | | |
| Computer use in 1997 | 0.460*** | 0.581*** | 0.430*** | -0.470*** | -1.209*** | -0.198*** |
|  | (0.051) | (0.055) | (0.084) | (0.050) | (0.175) | (0.048) |
| R squared | 0.508 | 0.535 | 0.258 | 0.455 | 0.412 | 0.185 |
| Levels in 2010 (O*NET) | | | | | | |
| Computer use in 2003 | 0.377*** | 0.396*** | 0.425*** | -0.525*** | -0.910*** | -0.321*** |
|  | (0.047) | (0.026) | (0.045) | (0.035) | (0.105) | (0.052) |
| R squared | 0.415 | 0.707 | 0.532 | 0.621 | 0.532 | 0.354 |
| Notes: N=322 occupations. OLS regression. weighted with employment weights. Robust standard errors are in parenthesis. * p<0.1, ** p<0.05, *** p<0.01. Computer use measured as the share of workers in the occupation that use computers on the job (source: CPS Computer Use Supplement October 1989, 1997, 2003). | | | | | | |

# Appendix B – Additional descriptive analysis

Table B1 reports the three transition matrices (decade-by-decade) for our set of 322 occupations, split by decade. Overall, we observe a relatively large number of shifts of occupations across different quintiles of RTI. Shifts affect 18% of occupations in the first decade (1980-1990), 56% of occupations in the second decade (1990-2000) and 48% of the occupations in the third decade (2000-2010). Interestingly, a relatively small share of occupations shifts by two or more quintiles: none in 1980-1990, 42 (13% of occupations) in 1990-2000, 21 (6% of occupations) in 2000-2010. Finally, we observe just 4 shifts of three quintiles (3 in 1990-2000 and 1 in 2000-2010) and no shift of four quintiles.

Table B1 – Transition matrix of RTI

|  |  | Quintile of RTI 1990 | | | | | |
| --- | --- | --- | --- | --- | --- | --- | --- |
|  |  | 1 | 2 | 3 | 4 | 5 | Total |
| Quintile of RTI 1980 | 1 | 58 | 7 | 0 | 0 | 0 | 65 |
|  | 2 | 11 | 50 | 3 | 0 | 0 | 64 |
|  | 3 | 0 | 3 | 53 | 8 | 0 | 64 |
|  | 4 | 0 | 0 | 8 | 47 | 9 | 64 |
|  | 5 | 0 | 0 | 0 | 9 | 56 | 65 |
|  | Total | 69 | 60 | 64 | 64 | 65 | 322 |
|  |  |  |  |  |  |  |  |
|  |  | Quintile of RTI 2000 | | | | | |
|  |  | 1 | 2 | 3 | 4 | 5 | Total |
| Quintile of RTI 1990 | 1 | 51 | 16 | 2 | 0 | 0 | 69 |
|  | 2 | 13 | 28 | 15 | 2 | 2 | 60 |
|  | 3 | 1 | 10 | 14 | 27 | 12 | 64 |
|  | 4 | 0 | 9 | 20 | 16 | 19 | 64 |
|  | 5 | 0 | 1 | 13 | 19 | 32 | 65 |
|  | Total | 65 | 64 | 64 | 64 | 65 | 322 |
|  |  |  |  |  |  |  |  |
|  |  | Quintile of RTI 2010 | | | | | |
|  |  | 1 | 2 | 3 | 4 | 5 | Total |
| Quintile of RTI 2000 | 1 | 50 | 13 | 2 | 0 | 0 | 65 |
|  | 2 | 15 | 31 | 15 | 2 | 1 | 64 |
|  | 3 | 0 | 17 | 23 | 15 | 9 | 64 |
|  | 4 | 0 | 3 | 20 | 24 | 17 | 64 |
|  | 5 | 0 | 0 | 4 | 23 | 38 | 65 |
|  | Total | 65 | 64 | 64 | 64 | 65 | 322 |
| Notes: Number of occupations by (unweighted) quintile of RTI in *t* and *t-10*. | | | | | | | |

We present here the full decomposition of the within and between components, using the following formula:

$\Delta RTI=\sum_{i,o} \left[ \bar{\delta_{i}\phi_{i,o}}\Delta RTI_{o}+\bar{\delta_{i}}\Delta\phi_{i,o}RTI_{o}^{1980}+\Delta\delta_{i}\bar{\phi_{io}}RTI_{o}^{1980}+\bar{\delta_{i}}\Delta\phi_{i,o}\left( \bar{RTI_{o}}-RTI_{o}^{1980} \right)+\Delta\delta_{i}\bar{\phi_{io}}\left( \bar{RTI_{o}}-RTI_{o}^{1980} \right) \right]$ (B.1)

where $\bar{\delta_{i}}\Delta\phi_{i,o}RTI_{o}^{1980}$ and $\Delta\delta_{i}\bar{\phi_{io}}RTI_{o}^{1980}$ are, respectively, the ‘pure’ between occupation and between industry components (calculated with the initial RTI). On the other hand, $\bar{\delta_{i}}\Delta\phi_{i,o}\left( \bar{RTI_{o}}-RTI_{o}^{1980} \right)$ and $\Delta\delta_{i}\bar{\phi_{io}}\left( \bar{RTI_{o}}-RTI_{o}^{1980} \right)$ represent the covariance components of, respectively, the between-occupation and between-industry components. As is evident from Table B2, the two covariance terms $\bar{\delta_{i}}\Delta\phi_{i,o}\left( \bar{RTI_{o}}-RTI_{o}^{1980} \right)$ and $\Delta\delta_{i}\bar{\phi_{io}}\left( \bar{RTI_{o}}-RTI_{o}^{1980} \right)$ appear relatively small compared to the other terms, so we focus on the simpler decomposition. Tables B3 and B4 replicate the full decomposition by macro-occupational and industry groups, respectively.

Table B2– Full decomposition of RTI

|  | 1980-1990 | 1990-2000 | 2000-2010 | 1980-2010 |
| --- | --- | --- | --- | --- |
| **Within occupation** | **0.022** | **-0.072** | **-0.011** | **-0.062** |
| *Between occupation (with* $RTI_{o}^{1980}$*)* | *-0.032* | *-0.022* | *-0.002* | *-0.057* |
| *Covariance between occupation* | *0.001* | *-0.003* | *-0.008* | *-0.010* |
| **Total between occupation** | **-0.032** | **-0.025** | **-0.011** | **-0.067** |
| *Between industry (with* $RTI_{o}^{1980}$*)* | *-0.020* | *-0.011* | *-0.013* | *-0.044* |
| *Covariance between industry* | *0.001* | *0.001* | *0.004* | *0.006* |
| **Total between industry** | **-0.018** | **-0.010** | **-0.009** | **-0.037** |
| **Total change** | **-0.028** | **-0.107** | **-0.031** | **-0.166** |
| Notes: Decomposition of RTI based on equations 2 (bold) and B.1 (italics). Weights are the product of Census (1980; 1990; 2000) sampling weights and annual hours of labour supply. | | | | |

Table B3– Full decomposition of RTI by decade and occupation

|  | 1980-1990 | 1990-2000 | 2000-2010 | 1980-2010 |
| --- | --- | --- | --- | --- |
|  | Abstract | | | |
| **Within occupation** | **0.026** | **-0.175** | **0.140** | **-0.009** |
| *Between occupation (with* $RTI_{o}^{1980}$*)* | *-0.002* | *-0.017* | *0.002* | *-0.017* |
| *Covariance between occupation* | *-0.001* | *0.013* | *-0.006* | *0.007* |
| **Total between occupation** | **-0.002** | **-0.004** | **-0.003** | **-0.010** |
| *Between industry (with* $RTI_{o}^{1980}$*)* | *-0.003* | *0.002* | *0.006* | *0.004* |
| *Covariance between industry* | *0.001* | *0.002* | *0.003* | *0.006* |
| **Total between industry** | **-0.002** | **0.003** | **0.009** | **0.010** |
| **Total change** | **0.022** | **-0.176** | **0.145** | **-0.009** |
|  | Clerical | | | |
| **Within occupation** | **0.012** | **-0.050** | **-0.0750** | **-0.113** |
| *Between occupation (with* $RTI_{o}^{1980}$*)* | *-0.028* | *-0.021* | *-0.002* | *-0.051* |
| *Covariance between occupation* | *-0.003* | *-0.010* | *-0.005* | *-0.017* |
| **Total between occupation** | **-0.031** | **-0.031** | **-0.007** | **-0.068** |
| *Between industry (with* $RTI_{o}^{1980}$*)* | *-0.008* | *-0.003* | *0.002* | *-0.008* |
| *Covariance between industry* | *0.001* | *0.002* | *-0.003* | *0.000* |
| **Total between industry** | **-0.007** | **-0.001** | **-0.001** | **-0.008** |
| **Total change** | **-0.026** | **-0.082** | **-0.082** | **-0.190** |
|  | Blue collar | | | |
| **Within occupation** | **0.010** | **0.026** | **-0.170** | **-0.134** |
| *Between occupation (with* $RTI_{o}^{1980}$*)* | *0.002* | *-0.003* | *0.026* | *0.025* |
| *Covariance between occupation* | *0.003* | *-0.008* | *-0.013* | *-0.018* |
| **Total between occupation** | **0.004** | **-0.011** | **0.013** | **0.006** |
| *Between industry (with* $RTI_{o}^{1980}$*)* | *-0.003* | *-0.008* | *-0.004* | *-0.015* |
| *Covariance between industry* | *0.001* | *-0.001* | *0.002* | *0.003* |
| **Total between industry** | **-0.002** | **-0.009** | **-0.001** | **-0.011** |
| **Total change** | **0.013** | **0.006** | **-0.158** | **-0.139** |
|  | Service | | | |
| **Within occupation** | **0.068** | **-0.081** | **0.018** | **0.004** |
| *Between occupation (with* $RTI_{o}^{1980}$*)* | *-0.010* | *-0.021* | *0.015* | *-0.016* |
| *Covariance between occupation* | *0.001* | *-0.002* | *-0.006* | *-0.007* |
| **Total between occupation** | **-0.009** | **-0.023** | **0.009** | **-0.023** |
| *Between industry (with* $RTI_{o}^{1980}$*)* | *-0.006* | *-0.010* | *-0.004* | *-0.021* |
| *Covariance between industry* | *0.002* | *-0.004* | *0.003* | *0.001* |
| **Total between industry** | **-0.005** | **-0.014** | **-0.001** | **-0.020** |
| **Total change** | **0.054** | **-0.118** | **0.026** | **-0.038** |
| Notes: Decomposition of RTI based on equations 2 (bold) and B.1 (italics). Macro-occupational groups defined in Table A3. Weights are the product of Census (1980; 1990; 2000) sampling weights and annual hours of labour supply. | | | | |

Table B4 – Full decomposition of RTI by decade and industry

|  | 1980-1990 | 1990-2000 | 2000-2010 | 1980-2010 |
| --- | --- | --- | --- | --- |
|  | Manufacturing industries | | | |
| **Within occupation** | **0.004** | **-0.054** | **-0.088** | **-0.138** |
| *Between occupation (with* $RTI_{o}^{1980}$*)* | *-0.026* | *-0.022* | *0.002* | *-0.046* |
| *Covariance between occupation* | *-0.021* | *-0.010* | *-0.033* | *-0.064* |
| **Total between occupation** | **-0.048** | **-0.032** | **-0.031** | **-0.110** |
| *Between industry (with* $RTI_{o}^{1980}$*)* | *-0.009* | *-0.008* | *-0.006* | *-0.023* |
| *Covariance between industry* | *-0.003* | *0.011* | *-0.006* | *0.002* |
| **Total between industry** | **-0.012** | **0.002** | **-0.012** | **-0.021** |
| **Total change** | **-0.055** | **-0.083** | **-0.131** | **-0.270** |
|  | Non-manufacturing industries | | | |
| **Within occupation** | **0.027** | **-0.076** | **0.003** | **-0.046** |
| *Between occupation (with* $RTI_{o}^{1980}$*)* | *-0.051* | *-0.022* | *-0.028* | *-0.102* |
| *Covariance between occupation* | *0.024* | *-0.001* | *0.021* | *0.045* |
| **Total between occupation** | **-0.027** | **-0.023** | **-0.007** | **-0.057** |
| *Between industry (with* $RTI_{o}^{1980}$*)* | *-0.013* | *0.003* | *-0.010* | *-0.019* |
| *Covariance between industry* | *0.004* | *-0.011* | *0.007* | *0.000* |
| **Total between industry** | **-0.009** | **-0.008** | **-0.003** | **-0.020** |
| **Total change** | **-0.008** | **-0.107** | **-0.007** | **-0.123** |
| Notes: Decomposition of RTI based on equations 2 (bold) and B.1 (italics). Macro-occupational groups defined in Table A3. Weights are the product of Census (1980; 1990; 2000) sampling weights and annual hours of labour supply. | | | | |

# Appendix C – Robustness checks

We report four robustness checks for our estimate of the link between RTI and occupational employment and wages.

As a first robustness check, we repeat our analysis by using a modified version of the RTI index which also accounts for the importance of non-routine manual tasks. This index, labelled as RTI*^NRM^*, is computed as follows:

$RTI_{o,t}^{NRM}=\log\left( \frac{{CLERIC}_{o,t}+{MANUAL}_{o,t}}{\frac{1}{2}{MATH}_{o,t}+{\frac{1}{2}LANG}_{o,t}+EYEHAND_{o,t}} \right)$ (C.1)

Results are reported in Tables C1 and C2. In general, while the results appear to be in line with our baseline results (Tables 7 and 10) in terms of sign and statistical significance, we observe that employment and wage changes appear to be more strongly correlated with the initial level of RTI compared to our baseline results. This difference, however, is generally small.

Table C1 – Robustness check: results for employment for RTI computed including non-routine manual task intensity (RTI*^NRM^*)

| Panel A - Only initial RTI*^NRM^* | | | | |
| --- | --- | --- | --- | --- |
|  | (1) | (2) | (3) | (4) |
| Dep: Δlog(Empl) | 1980-1990 | 1990-2000 | 2000-2010 | 1980-2010 |
| Initial RTI*^NRM^* | -0.298*** | -0.179** | -0.106** | -0.665*** |
|  | (0.080) | (0.088) | (0.043) | (0.149) |
| Offshorability | 0.670** | -0.391 | 0.062 | 0.613 |
|  | (0.280) | (0.350) | (0.188) | (0.525) |
| R sq | 0.319 | 0.179 | 0.382 | 0.370 |
| N | 29847 | 28897 | 28083 | 26531 |
| Panel B - Initial and change of RTI*^NRM^* | | | | |
|  | (1) | (2) | (3) | (4) |
| Dep: Δlog(Empl) | 1980-1990 | 1990-2000 | 2000-2010 | 1980-2010 |
| Initial RTI*^NRM^* | -0.275*** | -0.150* | -0.083* | -0.796*** |
|  | (0.097) | (0.083) | (0.049) | (0.155) |
| ΔRTI*^NRM^* | 0.291 | -0.307** | 0.048 | -0.322* |
|  | (0.348) | (0.129) | (0.069) | (0.189) |
| Offshorability | 0.714** | -0.544 | 0.065 | 0.524 |
|  | (0.283) | (0.360) | (0.184) | (0.550) |
| R sq | 0.321 | 0.188 | 0.382 | 0.372 |
| N | 29847 | 28897 | 28083 | 26531 |
| Notes: OLS model. All models include industry dummies. Weights are the product of Census (1980; 1990; 2000) sampling weights and annual hours of labour supply. Robust standard errors clustered at occupation level are in parenthesis. * p<0.1, ** p<0.05, *** p<0.01. In the stacked differences specification offshorability (unreported) and industry dummies are interacted with period dummies. RTI^NRM^ is defined as in equation D.1. | | | | |

Table C2 – Robustness check: results for wages for RTI computed including non-routine manual task intensity (RTI*^NRM^*)

| Panel A - Only initial RTI | | | | |
| --- | --- | --- | --- | --- |
| Dep: Δlog(average annual wage) | 1980-1990 | 1990-2000 | 2000-2010 | 1980-2010 |
| Initial RTI*^NRM^* | -0.0434* | -0.00157 | -0.0852*** | -0.221*** |
|  | (0.0248) | (0.0166) | (0.0110) | (0.0320) |
| Offshorability | -0.0673 | -0.0783 | 0.0566 | 0.0723 |
|  | (0.0650) | (0.0556) | (0.0683) | (0.0955) |
| R sq | 0.0372 | 0.0476 | 0.0382 | 0.0734 |
| N | 153792 | 172077 | 152197 | 108921 |
| Panel B - Initial and change of RTI | | | | |
| Dep: Δlog(average annual wage) | 1980-1990 | 1990-2000 | 2000-2010 | 1980-2010 |
| Initial RTI*^NRM^* | -0.0494* | -0.00308 | -0.153*** | -0.263*** |
|  | (0.0292) | (0.0178) | (0.0211) | (0.0351) |
| Change in RTI*^NRM^* | -0.0757 | 0.0107 | -0.136*** | -0.111*** |
|  | (0.0958) | (0.0256) | (0.0325) | (0.0361) |
| Offshorability | -0.0813 | -0.0726 | 0.0587 | 0.0428 |
|  | (0.0696) | (0.0615) | (0.0663) | (0.101) |
| R sq | 0.0373 | 0.0476 | 0.0399 | 0.0743 |
| N | 153792 | 172077 | 152197 | 108921 |
| Notes: OLS model. Unit of analysis: occupation, industry, gender, age (16-24, 25-39, 40-54, 55-65), education (less than high-school, high-school degree, college degree or more), year pairs. All models include industry dummies. Weights used are the start of period product of Census (1980; 1990; 2000) sampling weights and annual hours of labour supply. Robust standard errors clustered at occupation level in parenthesis. * p<0.1, ** p<0.05, *** p<0.01. | | | | |

Second, we repeat our analysis for a less demanding specification in which we exclude from the employment specification all occupation-specific control variables except RTI (i.e., Offshorability and Initial manual task intensity). Results are shown in Tables C3 and C4. Again, baseline results (Tables 7 and 10) are generally confirmed, with the only exception being a not significant link between initial RTI and employment change in Panel B for the decade 1990-2000.

Table C3 – Robustness check: results for employment without additional occupation-level control variables

| Panel A - Only initial RTI | | | | |
| --- | --- | --- | --- | --- |
| Dep: Δlog(Empl) | 1980-1990 | 1990-2000 | 2000-2010 | 1980-2010 |
| Initial RTI | -0.236*** | -0.116** | -0.075** | -0.473*** |
|  | (0.049) | (0.054) | (0.030) | (0.110) |
| R sq | 0.310 | 0.177 | 0.380 | 0.363 |
| N | 30015 | 29088 | 28083 | 26693 |
| Panel B - Initial and change of RTI | | | | |
| Dep: Δlog(Empl) | 1980-1990 | 1990-2000 | 2000-2010 | 1980-2010 |
| Initial RTI | -0.232*** | -0.088 | -0.058 | -0.598*** |
|  | (0.052) | (0.060) | (0.042) | (0.121) |
| ΔRTI | 0.191 | -0.213** | 0.040 | -0.402** |
|  | (0.248) | (0.106) | (0.082) | (0.194) |
| R sq | 0.311 | 0.183 | 0.380 | 0.366 |
| N | 30015 | 28897 | 28083 | 26531 |
| Notes: OLS model. All models include industry dummies. Weights are the product of Census (1980; 1990; 2000) sampling weights and annual hours of labour supply. Robust standard errors clustered at occupation level are in parenthesis. * p<0.1, ** p<0.05, *** p<0.01. | | | | |

Table C4 – Robustness check: results for wages without additional occupation-level control variables

| Panel A - Only initial RTI | | | | |
| --- | --- | --- | --- | --- |
| Dep: Δlog(average annual wage) | 1980-1990 | 1990-2000 | 2000-2010 | 1980-2010 |
| Initial RTI | -0.00315 | -0.0103 | -0.0781*** | -0.166*** |
|  | (0.0215) | (0.0126) | (0.00857) | (0.0263) |
| R sq | 0.0365 | 0.0473 | 0.0390 | 0.0727 |
| N | 154242 | 172705 | 152197 | 109291 |
| Panel B - Initial and change of RTI | | | | |
| Dep: Δlog(average annual wage) | 1980-1990 | 1990-2000 | 2000-2010 | 1980-2010 |
| Initial RTI | -0.00326 | -0.0134 | -0.137*** | -0.194*** |
|  | (0.0221) | (0.0121) | (0.0167) | (0.0285) |
| Change in RTI | -0.00649 | 0.0268 | -0.134*** | -0.0911** |
|  | (0.0731) | (0.0210) | (0.0291) | (0.0457) |
| R sq | 0.0365 | 0.0477 | 0.0407 | 0.0733 |
| N | 154242 | 172077 | 152197 | 108921 |
| Notes: OLS model. Unit of analysis: occupation, industry, gender, age (16-24, 25-39, 40-54, 55-65), education (less than high-school, high-school degree, college degree or more), year pairs. All models include industry dummies. Weights used are the start of period product of Census (1980; 1990; 2000) sampling weights and annual hours of labour supply. Robust standard errors clustered at occupation level in parenthesis. * p<0.1, ** p<0.05, *** p<0.01. | | | | |

Similarly to the estimates on the relationship between RTI and employment accounting for new jobs, we report the results of the estimates for wages controlling for new jobs (Table C5 first column) and shutting down the contribution of new jobs to within-occupation RTI change (Table C5 second column). Results show that including these additional controls does not influence our main results, which remain robust and in line with the ones of Table 10 in terms of sign, magnitude and statistical significance.

Table C5 – Estimates for wages accounting for new jobs

| Dep: Δlog(average annual wage), 1980-2010 | RTI (benchmark) | RTI (net of contribution of new jobs) |
| --- | --- | --- |
| Initial RTI | -0.209*** | -0.227*** |
|  | (0.0324) | (0.0321) |
| Change in RTI | -0.111*** | -0.139*** |
|  | (0.0427) | (0.0465) |
| Share of new jobs in occ | 0.00981 | -0.00225 |
|  | (0.0590) | (0.0559) |
| Offshorability | 0.172*** | 0.180*** |
|  | (0.0552) | (0.0554) |
| Initial NRM tasks | 0.000107 | 0.00723 |
|  | (0.101) | (0.0982) |
| R sq | 0.0742 | 0.0747 |
| N | 108884 | 108884 |
| Notes: OLS model. Unit of analysis: occupation, industry, gender, age (16-24, 25-39, 40-54, 55-65), education (less than high-school, high-school degree, college degree or more) pairs. All models include industry dummies. Weights used are the start of period product of Census (1980) sampling weights and annual hours of labour supply. Robust standard errors clustered at occupation level in parenthesis. * p<0.1, ** p<0.05, *** p<0.01. | | |

Finally, Tables C6 and C7 contain an exercise about the heterogenous effects of occupational groups and macro-sectors on wages (similarly to the one conducted on employment in Table 12 and 13 in the manuscript). Within-occupation task changes are particularly important in explaining wages of Abstract occupations and non-manufacturing sectors.

Table C6 – Estimates of wage change by macro-occupational group

| Dep: Δlog(average annual wage), 1980-2010 | Abstract occupations | Clerical occupations | Manual occupations | Service occupations |
| --- | --- | --- | --- | --- |
| Initial RTI | -0.213* | -0.257*** | -0.0654 | -0.0673 |
|  | (0.122) | (0.0751) | (0.0403) | (0.0546) |
| Change in RTI | -0.175* | 0.0887 | -0.0349 | -0.0700 |
|  | (0.0990) | (0.0738) | (0.0496) | (0.0740) |
| Offshorability | 0.179 | -0.130 | 0.0423 | 0.0403 |
|  | (0.125) | (0.264) | (0.0576) | (0.119) |
| Initial NRM tasks | -0.555* | 0.337** | 0.172 | -0.302 |
|  | (0.311) | (0.128) | (0.179) | (0.233) |
| R sq | 0.104 | 0.0528 | 0.0358 | 0.0985 |
| N | 31000 | 33566 | 33721 | 10464 |
| Notes: OLS model. Unit of analysis: occupation, industry, gender, age (16-24, 25-39, 40-54, 55-65), education (less than high-school, high-school degree, college degree or more) pairs All models include industry dummies. Weights used are the start of period product of Census (1980) sampling weights and annual hours of labour supply. Robust standard errors clustered at occupation level in parenthesis. * p<0.1, ** p<0.05, *** p<0.01. | | | | |

Table C7 – Estimates of wage change by macro-sector

| Dep: Δlog(average annual wage), 1980-2010 | Manufacturing sectors | Non-manufacturing sectors |
| --- | --- | --- |
| Initial RTI | -0.130*** | -0.228*** |
|  | (0.0340) | (0.0359) |
| Change in RTI | -0.0140 | -0.122** |
|  | (0.0671) | (0.0516) |
| Offshorability | -0.0994 | 0.234*** |
|  | (0.0745) | (0.0577) |
| Initial NRM tasks | 0.0880 | 0.0170 |
|  | (0.105) | (0.113) |
| R sq | 0.0328 | 0.0708 |
| N | 36581 | 72340 |
| Notes: OLS model. Unit of analysis: occupation, industry, gender, age (16-24, 25-39, 40-54, 55-65), education (less than high-school, high-school degree, college degree or more) pairs. All models include industry dummies. Weights used are the start of period product of Census (1980) sampling weights and annual hours of labour supply. Robust standard errors clustered at occupation level in parenthesis. * p<0.1, ** p<0.05, *** p<0.01. | | |
